# MSCs ameliorates hyperglycemia-induced endothelial injury through modulation of mitochondrial dynamics

**DOI:** 10.1038/s41419-025-08175-x

**Published:** 2025-11-17

**Authors:** Jingjing Wei, Ruiwen Mao, Yao Chen, Kunjie Si, Yao Li, Jiaqi Li, Wuzheng Zhu

**Affiliations:** 1 Hunan International Joint Laboratory of Animal Intestinal Ecology and Health, Laboratory of Animal Nutrition and Human Health, College of Life Sciences, Hunan Normal University, Changsha, PR China; 2 National Medical Metabolomics International Collaborative Research Center, Xiangya Hospital, Central South University, Changsha, PR China; 3 National Clinical Research Center for Geriatric Disorders, Xiangya Hospital, Central South University, Changsha, PR China

**Keywords:** Mesenchymal stem cells, Vascular diseases, Apoptosis

## Abstract

Endothelial dysfunction contributes to the development of cardiovascular disease in patients with diabetes mellitus, and current strategies remain inadequate. Although mesenchymal stromal cells (MSCs) have shown beneficial effects in experimental models of diabetes, underlying mechanisms remain elusive. Here, using a human umbilical vein endothelial cells (HUVECs) treated with high concentration of glucose (HG) and a mouse model of type 2 diabetes (db/db mice), we demonstrate MSCs could alleviate hyperglycemia-induced endothelial injury by preventing aberrant mitochondrial morphology. Mechanistically, stanniocalcin-1(STC1) was identified to be an important paracrine factor secreted by MSCs, which restrains hyperactivation of ERK1/2, thus preventing Drp1-mediated excessive mitochondrial fission, and thereby protecting against hyperglycemia-induced oxidative injury, endothelial inflammation and mitochondrial apoptotic pathway, consequently protecting endothelial dysfunction. Hence, this study reveals that MSCs-derived STC1 regulates mitochondrial dynamics remodeling through inhibiting ERK1/2-Drp1 axis and provide a therapeutic target in diabetic vasculopathy and regenerative medicine.

## Introduction

Diabetes mellitus is the most common metabolic disorder in the world. Cardiovascular complications, such as myocardial infarction, stroke, diabetic nephropathy, and atherosclerosis, are the main cause of death from diabetes mellitus [1, 2]. A precursor to the development of diabetic vasculopathy is endothelial dysfunction, characterized by increased oxidative stress, endothelial inflammation and impaired endothelium-dependent vascular relaxation [3, 4]. Hyperglycemia-induced reactive oxygen species (ROS) has been demonstrated to be the main culprit for endothelial dysfunction in diabetes [5]. An increased level of ROS can directly cause cell injury by oxidative stress and decreased nitric oxide (NO) bioavailability, which in turn induces expression of vascular proinflammatory genes [6, 7]. Although significant advances have been made in our understanding of the pathobiology and progression of diabetic vasculopathy, current treatment options for diabetic vasculopathy remain limited, and new and effective therapeutic approaches are urgently needed.

Mitochondrial damage has been recognized as a common early event preceding cell functional loss and death, which is inextricably linked to the pathogenesis of a wide range of microvascular and macrovascular complications of diabetes. Evidence to date indicates that mitochondria are the major source of ROS in hyperglycemia, as HG increases metabolic input into mitochondria and overwhelms the electron transport system, resulting in ROS overproduction [8]. In turn, the burst of mitochondrial ROS (mtROS) further accelerates oxidative damage to mitochondrial components and thus initiates a cycle of positive feedback, resulting in structural disruption and functional impairment of mitochondria, triggering the release of proapoptotic proteins like cytochrome C to induce cell death, and mitochondrial DNA (mtDNA), which can serve as a proinflammatory danger signal. Given this, increased mtROS production has been postulated as the central hub for several key pathogenic pathways involved in diabetic vasculopathy [5]. However, the precise mechanisms underlying the molecular cross-talk between mitochondrial damage and endothelial dysfunction in diabetic conditions have not yet been fully elucidated.

Mitochondria are dynamic organelles that frequently undergo fission and fusion process to maintain mitochondrial integrity and function as well as cellular homeostasis. Mitochondrial fusion facilitates the exchange of metabolites, substrates, and mtDNA between mitochondria to ensure optimal function of the mitochondrial network, which is beneficial for the complementation of oxidatively damaged mitochondrial components to mitigate organelle stress. Fission, under physiologic conditions, is essential for the mitotic segregation of mitochondria into daughter cells and the selective removal of damaged or dysfunctional components of mitochondria via mitophagy. It is noteworthy, however, that excessive mitochondrial fission may be detrimental, as it not only induces progressive energy deficiency but also facilitates mitochondrial membrane leakage and consequent cell death. Exposure of cultured endothelial cells or isolated arterial tissue to HG environment caused mitochondrial fission [3, 9]. More specifically, mitochondrial fragmentation mediated by fission has been documented as an early event prior to ROS overproduction and cellular apoptosis under hyperglycemic conditions [10]. Additionally, accumulating evidence suggests that imbalanced mitochondrial dynamics contribute critically to the pathogenesis diabetic vasculopathy, in which excessive fission mediates endothelial pathologies, including impairment of endothelium-dependent relaxation, an increase in endothelial inflammation and defects in wound healing and angiogenesis [3, 11]. On the basis of these data, it appears that mitochondrial dynamics could potentially control ROS overproduction in diabetes and confer resistant to hyperglycemic-induced endothelial injury.

Mesenchymal stromal cells (MSCs), identified as multipotent adult stem cells, have emerged as a promising candidate for cell-based therapy in a diverse range of diseases duo to their regenerative capability, anti-inflammatory and immunosuppressive properties [12, 13]. The homeostatic functions of MSCs have formerly been explained by their capacity for multilineage differentiation and cell–cell interactions [14]. However, there is an increasing recognition that the primary mechanism underlying their therapeutic action is predominantly paracrine in nature, involving the secretion of soluble factors or release of extracellular vesicles. Thus far, a large number of preclinical and clinical studies have seen an inspiring therapeutic effect of MSCs in various degenerative and inflammatory disorders, including diabetes mellitus [12, 15, 16]. Co-transplantation of human MSCs with porcine islets was reported to have a greater cellular insulin content and increased glucose-stimulated insulin secretion in a diabetic mouse model due to earlier islet vascularization and improved islet engraftment [17]. Recent evidence reveals that adipose tissue-derived MSCs could ameliorate HG-induced endothelial dysfunction by suppressing oxidative stress and concomitant proinflammatory phenotypes [18]. In addition, we previously found that MSCs treatment can maintain correct mitochondrial morphology and function in response to HG insults and protect endothelial cells against the mitochondrial apoptotic pathway. Nevertheless, the precise endothelial targets responsible for the observed therapeutic effects of MSC remain enigmatic, and the most effective paracrine factors secreted by MSCs to maintain mitochondrial fitness have not been fully identified.

In the present study, we found that MSCs infusion could inhibit endothelial injury in diabetic mice by preventing hyperglycemia-induced mitochondrial fission, oxidative damage, and resultant inflammation. Furthermore, STC1 was identified as the effective mediator secreted by MSCs to suppress ERK1/2-Drp1 axis-mediated excessive fission.

## Methods and materials

### Cell isolation and culture

All procedures involving human subjects were performed following the Declaration of Helsinki and were approved by the Ethics Committee of Hunan Normal University and the First Affiliated Hospital of Hunan Normal University. Informed consent was obtained from the donator of samples. Human umbilical vein endothelial cell (HUVECs) were isolated from human umbilical cord veins of newborns and identified by flow cytometry as previously described. [19, 20] Endothelial cells were cultured in supplemented ECM-2 basal media (Millipore Corporation, Billerica, MA, USA) in a humidified atmosphere of 5% CO_2_/95% air at 37 °C. As control cells, human skin fibroblast cells (HSFs) were originally isolated by collagenase digestion of human newborn foreskin as previously described and maintained in DMEM (Gibco, Grand Island, NY, USA) containing 10% FBS (Gibco) [21, 22]. Human Umbilical Cord-MSCs (hMSCs) were provided by Sichuan Neo-Life Stem Cell Biotech Inc. and cultured in low-glucose DMEM (Gibco, Grand Island, NY, USA) containing 10% FBS (Gibco) as previously described [23]. Cells between passages 3 and 6 were used in all experiments reported herein. Human monocyte cell line (THP1, ATCC TIB202) was cultured in RPMI 1640 medium supplemented with 10% fetal bovine serum.

### Coculture and treatment

The effects of HG on endothelial cell function were investigated by incubating with control (5.5 mmol/L) or HG (30 mmol/L) medium as previously described [3]. For coculture experiments, hMSCs or HSFs were seeded in a transwell insert at a density of 1.0 × 10^4^/cm^2^ and cultured in complete low-glucose DMEM medium for 12 h before coculture with HUVECs. Prior to coculture, hMSCs or HSFs were washed with PBS three times and cocultured with HUVECs (ratio 1:3) in ECM-2 media with a final glucose concentration of 30 mmol/L.

### Transfection experiments

For siRNA transfection, cells were transfected specific siRNA targeting HGF, IGF-1, SDF1, STC1, ANGPTL4, Galectin-1, IL-10, IL-6, TGF-β1, IDO1, COX-2, TSG-6, ERK1, ERK2, and their negative control siRNA using a commercial kit (JetPRIME^®^ transfection reagent, polyplus, USA). The sequences of primers are listed in the Supplemental Data. For plasmid DNA transfection, pCMV-ERK2, pCMV-Drp1S616D and pCMV-Drp1K38A plasmids were constructed by Shanghai GeneChem Co., Ltd. (Shanghai, China). Cells were transfected with DNA (1–3 μg) using a commercial kit (JetPRIME^®^ transfection reagent, polyplus, USA). After 24–48 h incubation, the transfection mixture was removed and replaced with fresh complete medium. Thereafter, cells were used for further experiments.

### ELISA

STC1 measurements in the media were conducted according to the manufacturer’s instructions using the STC1 ELISA kit (R&D Systems) in cell culture supernatant and mouse serum.

### Monocyte adhesion assay

Human peripheral blood mononuclear leukocytes (THP-1 cells) were cultured in RPMI 1640 medium (Gibco) with 10% fetal bovine serum, as suspension culture, and were labeled with CM-Dil (ThermoFischer Scientific, 1 μg/mL) in RPMI media without serum at a concentration of 5 μM at 37 °C for 30 min, followed by wash in phosphate-buffered saline (PBS) for 3 times. Resuspend CM-Dil-labeled THP-1 cell pellet in serum free 1640 medium and adjust the cell concentration with media to 2 × 10^6^ cells/mL. Add 0.5 mL (1 × 10^6^ cells) of THP-1 cell suspension into each well of the 12-well cluster plate containing the treated HUVECs monolayer and incubate the plate in a cell culture incubator at 37 °C and 5% CO_2_ for 1–3 h. Followed by PBS wash for 3 times, the adherent monocytes were fixed with 4% paraformaldehyde for 10 min at room temperature. Cells were counted in 10 randomly selected microscopic fields under an inverted epi-fluorescence microscope.

### Immunofluorescence

Cells were washed in PBS, fixed with 4% paraformaldehyde in PBS for 10 min, permeabilized with 0.1% Triton X-100 in PBS for 10 min, blocked in 1% BSA in PBS for 30 min, and incubated with primary antibody COXⅣ (1:300, Cat# 11967, Cell Signaling Technology) for overnight in 4 °C, followed by Goat anti-mouse Alexa Fluor 488 secondary antibody (1:500, Cat# A-21121, Cell Signaling Technology) for 1 h at room temperature. Followed by PBS wash for 3 times, cells were mounted onto coverslips covered in Fluoromount-G™ Mountant with DAPI (eBioscience).

### Mitochondrial morphology assessment

Mitochondria morphology was detected by immunofluorescence staining with COXⅣand examined by confocal microscopy (Nikon A1, Nikon Corporation, Japan). Investigators were not blinded to allocation during the experiments. Form factor (FF) was defined as (perimeter^2^/4*π*·area). Aspect ratio (AR) was determined as the ratio between the major and minor axes of the ellipse equivalent to the mitochondrion. Both parameters have a minimal value of 1 if the particle is a small perfect circle and the values increase as it becomes longer. AR represents mitochondrial length and increases in form factor indicate increases in mitochondrial complexity. Images were quantitated and analyzed by Image-Pro Plus software.

### Mitochondrial membrane potential

Cells were washed with PBS, then collected by trypsinization, and loaded with 50 nM TMRM probe (Cat# T-668, ThermoFischer Scientific) for 30 min at 37 °C, after which the cells were washed twice with PBS again and resuspended in PBS, filtered and subjected to flow cytometry (Moflo Astrios EQ, Beckman Coulter) analysis. Flow cytometry data were recorded for 10,000 events. Fluorescence intensity data was collected using excitation 561 nm, emission 579 ± 16 nm.

### Measurements of mitochondrial ROS and ATP production

MtROS production was measured with a flow cytometer. Cells were washed twice with PBS and loaded with 5 µM MitoSOX (Cat# M36009, ThermoFischer Scientific) for 30 min, after which the cells were washed twice with PBS and analyzed with flow cytometry (Moflo Astrios EQ, Beckman Coulter). ATP levels were measured using the ATP Assay Kit (Beyotime Biotechnology) according to the manufacturer’s instructions.

### Cell apoptosis assay

Cell apoptosis was analyzed using an Annexin V-FITC/PI staining kit (BD Biosciences, USA). After treatment, the cells were collected and labeled for 15 min with Annexin V and propidium iodide (PI). The apoptotic cells were detected via flow cytometry (Moflo Astrios EQ, Beckman Coulter).

### Flow cytometric analyses of isolated mitochondria

Isolated aorta mitochondria were stained with 100 nM MitoTracker Green (Cat# M46750, ThermoFischer Scientific) and 150 nM MitoTracker Deep Red (Cat# M22426, ThermoFischer Scientific) at room temperature for 20 min and washed twice with PBS. Mitochondrial status was determined by flow cytometric (Moflo Astrios EQ, Beckman Coulter).

### Quantitative Real Time RT-PCR (qPCR)

Quantitative real time PCR amplification was conducted with QuantStudio5 (Applied Biosystems) using Power SYBR Green PCR Master Mix (Applied Biosystems) according to the protocol recommended by the manufacturer. The cycling program was 95 °C for 5 min to activate the polymerase followed by 40 cycles of denaturation at 95 °C for 15 s, annealing at 60 °C for 30 s and extension at 72 °C for 30 s. All reactions were performed in triplicate for each cDNA sample. In addition, no template controls were tested for each primer pair. Data were analyzed using the 2^−ΔΔCt^ method and presented as fold changes relative to control. The sequences of primers are listed in the Supplemental Data.

### Mitochondrial isolation

Mitochondria were isolated from HUVECs and aorta tissues using the Cell Mitochondria Isolation Kit (Beyotime Institute of Biotechnology, China) and the Tissue Mitochondria Isolation Kit (Beyotime Institute of Biotechnology, China), respectively. Briefly, samples were placed in ice-cold mitochondria isolation buffer and homogenized using a Douce homogenizer (Kontes Glass Co.) The homogenate was centrifuged at 600 × *g* for 10 min at 4 °C. The supernatant was collected and then centrifuged at 11,000 × *g* for 10 min at 4 °C to pellet the mitochondria, which were resuspended for analyses. After mitochondria collection, the supernatants were collected and centrifuged again at 12,000 × *g* for 10 min at 4 °C. The supernatants were collected as the cytosol fraction. The total protein concentration of the isolated mitochondria and cytosol fraction was determined by the BCA Protein Assay Kit (Beyotime Biotechnology).

### Isolation of bone marrow MSCs

To generate bone marrow MSCs, bone marrow mononuclear cells were harvested by flushing the tibiae and femurs of 3-week-old male mice with saline. Cells were cultured in low glucose DMEM containing 10% FBS at 37 °C in 5% humidified CO_2_ and identified as previously described [24]. MSCs between passages three and four were used for transplantation.

### Animal experiments

All animal experiments were approved by the Institutional Animal Care and Use Committee (IACUC) of Hunan Normal University (approval number: HNNU2022-185). The sample size for the animal studies was calculated based on a survey of data from published research or preliminary studies. Diabetic Lepr^*db*/*db*^ (*db*/*db*) and non-diabetic heterozygous Lepr^*db*/+^ (*db*/m) mice were purchased from SJA Laboratory Animal Corporation (Hunan, China). Mice were housed in a room at a constant temperature of 22 °C ± 2 °C with 12-h light-dark cycles, and were allowed free access to water and food. All animals were randomized before treatment. To investigate the effect of mice MSC treatment in diabetes, twelve-week-old male *db/db* mice were injected with bone marrow-derived MSC for a total of 12 weeks. MSCs (1 × 10^6^ per mouse; *n* = 6) at fifth passage were resuspended in 150 µL of 0.9% saline and delivered via tail-vein injection once a week using a 27 G needle. The same volume of saline was injected into control mice (*n* = 6). No mice were excluded from the statistical analysis. For RNAi experiments, MSCs were transfected with siRNA for STC1 using JetPRIME (Polyplus, USA) according to the protocol supplied by the manufacturer. The successful knockdown of STC1 expression was confirmed by real-time RT-PCR, followed by injection of the cells into mice. For tissue isolations, mice were anaesthetized with 2% isoflurane inhalation. Mice were euthanized by cervical dislocation under isoflurane anesthesia. All animals were treated in a blinded fashion, as the MSCs used for treating animals were prepared by researchers who did not carry out the treatments.

### Western blot analysis

HUVECs and aorta tissues were lysed in RIPA Buffer. Lysates were cleared by centrifugation at 15,000 r.p.m. for 15 min at 4 °C, and protein concentrations were measured by the BCA Protein Assay Kit (Beyotime Biotechnology). Aliquots of cell extracts containing 20–60 mg of protein were prepared in SDS-sample buffer, subjected to SDS-PAGE and transferred to polyvinylidene difluoride membranes (Millipore Corporation, Billerica, MA, USA). After blocking with 5% nonfat milk for 1 h, membranes were probed with the following primary antibodies overnight at 4 °C: Drp1 (1:1000, Cat# 61112) was purchased from BD Bioscience. ERK1/2 (1:1000, Ca# 11257-1-AP); Phospho-ERK1/2 (1:1000, Ca# 28733-1-AP); Fis1 (1:1000, Ca# 10956-1-AP) and ICAM1(1:1000,16174-1-AP) were purchased from Proteintech. OPA1 (1:1000, Ca# 67589); MFN2 (1:1000 Ca# 9482); Tom20 (1:1000 Cat# 42406); Bax (1:1000 Cat# 2772,); Cleaved Caspase-3 (1:1000, Cat# 9664); Caspase-3 (1:1000, Cat# 9662); phospho-Drp1 (Ser616) (1:1000, Cat# 63940); phospho-Drp1 (Ser637) (1:1000, Cat# 4867); COXⅣ (1:1000, Cat# 11967) and Cytochrome C (1:1000, Cat# 4272) were purchased from Cell Signaling Technology. VCAM1 (1:1000, Cat# A0279); β-actin (1:5000, Cat# AC006) and Bcl-2 (1:500, Cat# A2212) were obtained from Abclonal. Afterward, the membranes were probed with a HRP-conjugated secondary antibody (1:1000, Cat# AS014, Abclonal; 1:1000, Cat# AS003, Abclonal; 1:1000, Cat# 98164, Cell Signaling Technology) for 1 h at room temperature. Then, the proteins were visualized by enhanced chemiluminescence (ECL; Amersham, UK) reagents in the Molecular Imager Gel Doc XR System (Bio-Rad, Hertfordshire, UK). The blots were analyzed with densitometry using Image J software (NIH, Bethesda, MD, USA).

### Immunoprecipitation

Aorta tissues were homogenized and resuspended in NP-40 lysis buffer (20 mM Tris-HCl at pH 7.4, 150 mM NaCl, 1 mM EDTA, 1% NP-40, 10% glycerol, and protease inhibitor cocktail) and incubated at 4 °C for 30 min, followed by centrifugation at 12,000 × *g* and 4 °C for 10 min. The supernatants were pre-cleared with protein A/G magnetic beads. After the centrifugation, supernatants were incubated with either the indicated primary antibody or isotype-matched IgG at 4 °C overnight. Then the immunoprecipitated complexes were collected by incubation with 20 µL of protein A/G magnetic beads (Bimake) at 4 °C for 4 h. The beads were washed three times with washing buffer (50 mM Tris-HCl at pH 7.4, 150 mM NaCl, 0.1% Triton X-100 and protease inhibitor cocktail), eluted in 1 × SDS buffer (50 mM Tris-HCl at pH 6.8, 2% SDS, 0.01% bromophenol blue, 10% glycerol, 1% 2-mercaptoethanol and protease inhibitor cocktail) and boiled for 10 min. The immunoprecipitates were detected by Western blot.

### Drp1 GTPase activity assay

Cells were lysed in lysis buffer (50 nM HEPES, pH 7.5, 120 mM NaCl, 5 mM EDTA,10 mM Na pyrophosphate, 50 mM NaF, 1 mM Na_3_VO_4_, 1% Triton X-100, protease inhibitors) and total cell lysate (1 mg) was used for immunoprecipitation using Drp1 antibody (BD Bioscience) for overnight at 4 °C. Anti-Drp1 immunoprecipitates were incubated with 50 μL protein A/G magnetic beads (Bimake) for 2 h, and beads were washed three times with lysis buffer, and further washed three times with GTPase buffer (50 mM Tris, pH 7.5, 2.5 mM MgCl_2_, 0.02% β-Mercaptoethanol) as described previously [11]. The GTPase activity was measured using the GTPase assay kit by following manufacturer’s instruction (Innova Bioscience).

### Immunohistochemistry

After dewaxing and rehydration, 5-mm-thick aorta sections were subjected to heat–induced epitope retrieval in Tris-EDTA Buffer (10 mM Tris Base, 1 mM EDTA Solution, and 0.05% Tween 20, pH 9.0). Nonspecific interactions were blocked with 10% Normal Donkey Serum in 1 × Tris-Buffered Saline with Tween for 1 h at room temperature. Sections were then incubated with anti- phospho-Drp1(Ser616) (Cat# 63940, Cell Signaling Technology), Phospho-ERK1/2 (Ca# 28733-1-AP, Proteintech) antibodies at 4 °C overnight in blocking solution. The next day, sections were washed in Tris-Buffered Saline with Tween and treated with a 3% H_2_O_2_ solution to reduce endogenous peroxidase activity followed by incubation with secondary antibodies (Zhongshan Biotech, Beijing, China). Signals of the antigen-antibody complexes were visualized with Vectastain^®^ ABC Standard kit (PK-4000, Vector Laboratories) following the protocol of the manufacturer.

### Transmission electron microscopy

Freshly isolated aortas were cut into 1-mm aortic rings, washed in 0.1 M phosphate buffer, and fixed in 2.5% glutaraldehyde solution and then dehydrated and embedded in Epon resin. Ultra-thin sections of embedded tissues were stained with 5% uranyl acetate and lead citrate solution and analyzed by TEM. The mitochondrial AR (major axis/minor axis) was determined by Image-Pro Plus software.

### Vascular mtROS and NO production assays

The in situ production of mtROS and NO was measured using the fluorescent dyes MitoSOX Red (Ca# M36009, ThermoFischer Scientific) and 4-Amino-5-Methylamino-2′,7′-114 Difluorofluorescein (DAF-FM) (Ca# D23841, ThermoFischer Scientific), respectively. Mice were anesthetized with pentobarbital sodium (50 mg/kg body weight). Then, one of the femoral arteries was cut to drain blood, and the circulatory system was perfused with saline containing 40 U/mL heparin through the left ventricle until the saline flowing out from the cut become clear. Subsequently, aortas were removed, stripped of fat and connective tissues carefully, cut open longitudinally. Isolated segment vessels were cut into 2–3-mm-wide sections and placed on a glass slide to flatten down the aorta with the endothelial surface facing upwards. Then Krebs-HEPES buffer containing 2 μM MitoSOX Red or 5 μM DAF-FM was then applied to each section and incubated at 37 °C for 30 min. Aorta segments were then mounted with Fluoromount-G™ fluorescence mounting media containing DAPI (eBioscience). Fluorescence intensity was evaluated under fluorescence microscopy.

### Measurement of endothelial function

The aorta was quickly removed and dissected from adhering connective tissue. Aortas were cut into 3-mm rings, with special care taken to preserve the endothelium, and mounted in organ baths filled with Krebs-Ringer bicarbonate solution (118.3 mmol/L NaCl, 4.7 mmol/L KCl, 2.5 mmol/L CaCl_2_, 1.2 mmol/L MgSO_4_, 1.2 mmol/L KH_2_PO_4_, 25 mmol/L NaHCO_3_, 5.5 mmol/L D-glucose) aerated with 95% O_2_ and 5% CO_2_ at 37 °C. The rings of the aorta were attached to a force transducer, and isometric tension was recorded using DMT wire myograph systems (DMT 620 M, Denmark). The rings were primed with 30 mmol/L KCl and then precontracted with norepinephrine (1 μM, Sigma), producing a submaximal contraction. After the plateau was attained, the rings were exposed to the cumulative addition of 10^−9^–10^−5^ mol/L acetylcholine (Sigma) or 10^−9^–10^−5^ mol/L sodium nitroprusside (Sigma). Changes in the tension of the aortic rings were measured. To confirm that a possible recovery of endothelial function after MSCs treatment was due to the improved mitochondrial dynamics, aortas were preincubated with PD980591(Ca# S1177, Selleck) or Mdivi-1(Ca# S7162, Selleck) for 6 h at 37 °C before experiments.

### Seahorse metabolic analyzer assays

HUVECs in equal numbers were seeded into a XFe96 cell culture plates (Seahorse Bioscience, Billerica, MA) at a cell density of 2 × 10^4^ cells per well and allowed to adhere and grow for 24 h in a 37 °C humidified incubator with 5% CO_2_. Cells were washed with the Seahorse XF Base Medium (unbuffered DMEM,1 mM sodium pyruvate, 2 mM glutamine, and 10 mM glucose) and incubated for 45 min in a 37 °C without CO_2_ before starting the assay. Samples were then run on a Seahorse XFe96 Extracellular Flux Analyzer (Agilent Technologies) using the Seahorse XF Cell Mito Stress Test kit with 1 μM oligomycin, 1 μM carbonyl cyanide-4-(trifluoromethoxy) phenylhydrazone (FCCP), and 0.5 μM rotenone/antimycin A. All samples were run in triplicate.

### Data and statistical analysis

Statistically significant differences between means of two groups were analyzed by using Student’s *t* test where appropriate, or one-way ANOVA followed by Tukey’s test for comparisons involving more than two groups. Statistical analyses were performed using Prism 8 (GraphPad). *P* < 0.05 was defined as statistical significance. Sample size was chosen empirically based on our previous experience in the calculation of experimental variability.

## Results

### MSCs ameliorate HG-induced mitochondrial fragmentation, mtROS elevation and endothelial inflammation

**Fig. 1:**
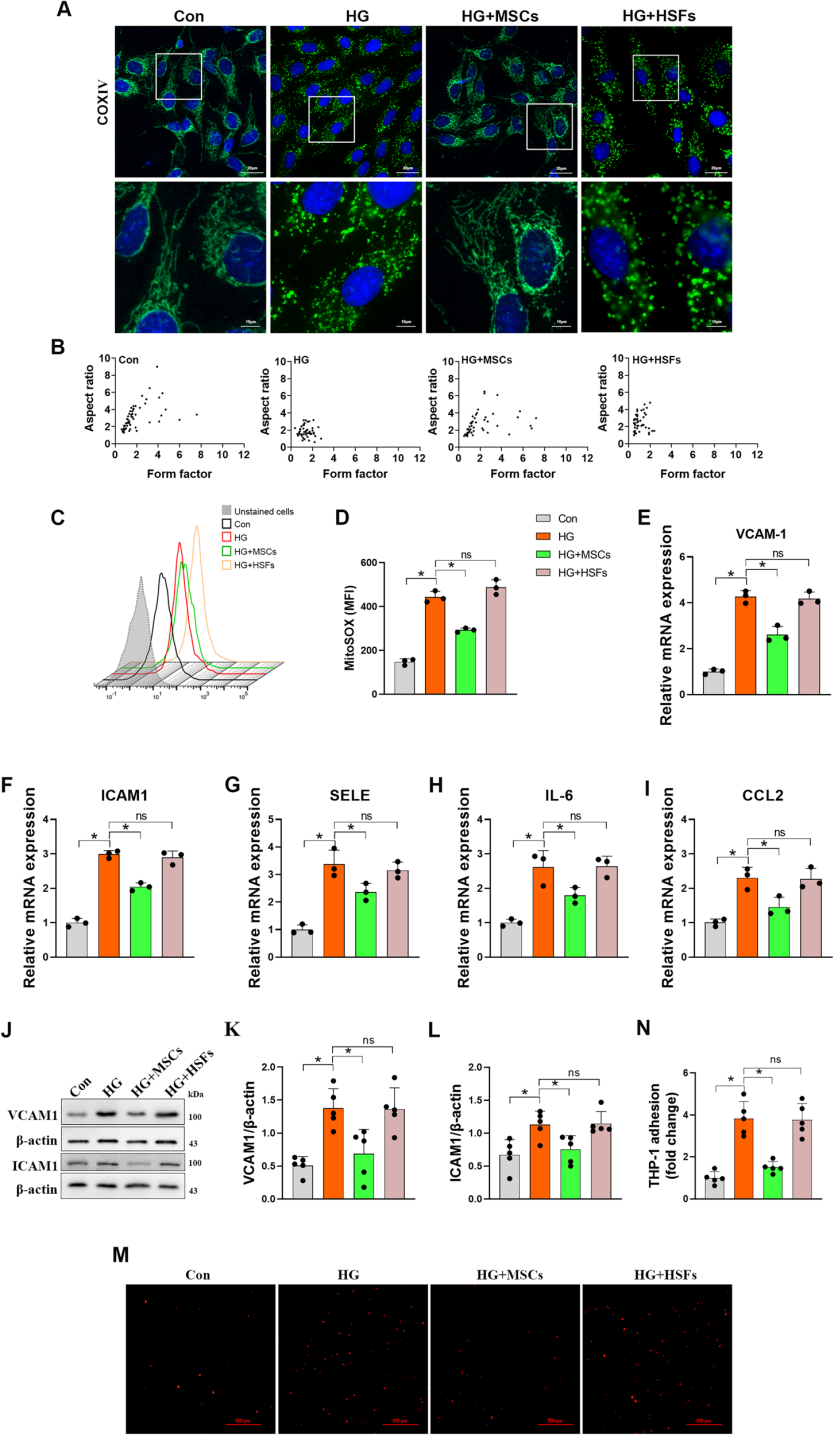
MSCs coculture inhibits HG-induced mitochondrial fragmentation, mtROS overproduction and endothelial inflammation. **A** Representative images of immunofluorescence analysis of COXIV-labeled mitochondria in HUVECs. **B** Aspect ratio and form factor were quantified for each group (*n* = 60–100 cells/group). **C**, **D** Mitochondrial ROS measurements described as mean fluorescence intensity (MFI) of MitoSOX (*n* = 3 independent experiments). **E**–**I** mRNA expression of inflammatory adhesion molecules in HUVECs (*n* = 3 independent experiments). **J**–**L** Representative Western blots and densitometric analysis of protein levels of ICAM1 (intercellular adhesion molecule 1) and VCAM1 (vascular cell adhesion molecule 1) (*n* = 5 independent experiments). **M**, **N** Representative images and corresponding quantitation showed the adhesion of monocytes to the lawn of HUVECs (*n* = 5 independent experiments). Data are shown as means ± SD. ns indicates not significant. *P < 0.05. Data were analyzed using one-way ANOVA with Tukey’s post hoc test.

We stimulated HUVECs with HG as previously described [19] and performed immunofluorescence staining for COXIV, a mitochondrial marker. Cells from control groups displayed elongated, thread-like mitochondria in complex networks, whereas fragmented phenotype with higher proportion of smaller punctate mitochondria was observed upon shifting to HG concentrations (Fig. 1A). Importantly, coculture with MSCs rescued mitochondrial morphology in HUVECs exposed to HG conditions, which mainly resembled normal glucose conditions. Indeed, when we quantified changes in mitochondrial morphology by mitochondrial AR and form factor (AR), we found both parameters were markedly improved in HUVECs cocultured with MSCs compared with HG alone (Fig. 1B). However, this favorable phenotype was not observed when cocultured with HSFs. Since mitochondrial fragmentation has been confirmed to be a critical factor contributing to increased ROS levels in HG conditions, we next determined whether MSCs administration could attenuate HG-induced mtROS overproduction. mtROS was detected by mitochondria-specific ROS indicator MitoSOX. MitoSOX fluorescence in the HG group was increased compared with the control group, but it was significantly normalized when cocultured with MSCs (Fig. 1C, D). Sustained oxidative stress can result in chronic inflammation in many cell types [25]. Thus, we detected the expression of proinflammatory genes in HUVECs. The mRNA level of vascular adhesion molecules *VCAM1*, *ICAM1*, and *SELE*, chemokine *CCL2* and cytokine *IL-6* was all upregulated upon HG exposure (Fig. 1E–I), indicating a proinflammatory phenotype of endothelial cell. Coculture with MSCs reduced the expression of these proinflammatory genes. Accordingly, consistent results were also observed in immunoblotting assays (Fig. 1J–L). Furthermore, monocyte attachment assay showed that MSCs treatment inhibits HG-induced adhesion of monocytes to HUVECs (Fig. 1M, N). These results imply that improvement of mitochondrial morphology mediated by MSCs may be associated with, and likely causative for, suppression of oxidative stress and inflammatory response in endothelial cells.

### MSCs mitigates HG-induced fragmentation by decreasing Drp1-dependent mitochondrial fission

**Fig. 2:**
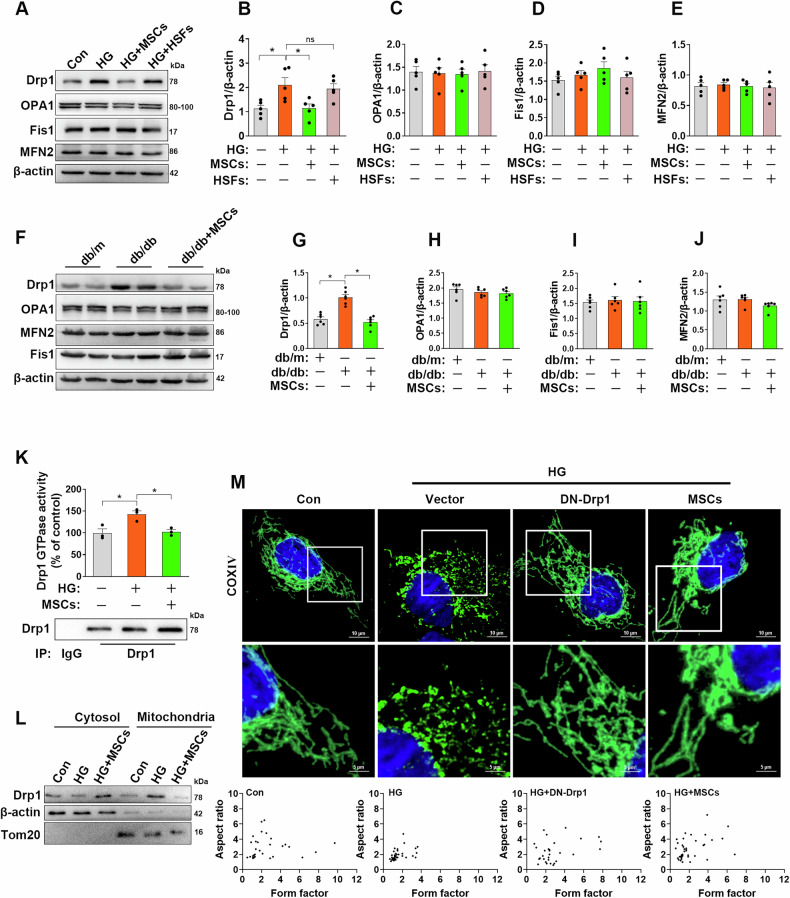
MSCs inhibits HG-induced mitochondrial fragmentation in a Drp1-dependent manner. **A**–**E** Mitochondrial dynamics-related proteins, including Drp1, Fis1, MFN2, and OPA1 in the HUVECs were analyzed by Western blots (*n* = 5 independent experiments). **F**–**J** Representative Western blots and densitometric analysis of Drp1, Fis1, MFN2 and OPA1 protein expression in mouse aortas (*n* = 6 mice/group). **K** Lysates immunoprecipitated with anti-Drp1 antibody were used to measure Drp1 GTPase enzyme activity (*n* = 3 independent experiments). **L** Cytoplasmic and mitochondrial fractions from HUVECs were analyzed by Western blots, the fractions were probed for Drp1. **M** HUVECs were transfected with DN-Drp1, empty vector or cocultured with MSCs, cells were then incubated with HG for 48 h, micrographs of mitochondrial morphology were visualized by anti-COXIV antibody staining (*n* = 60–100 cells/group). Data are shown as means ± SD. ns indicates not significant. *P < 0.05. Data were analyzed using one-way ANOVA with Tukey’s post hoc test.

Balanced frequency of fission and fusion determines mitochondrial morphology. To gain insight into the mechanism by which MSCs inhibit mitochondrial fragmentation, we examined the expression of key mitochondrial factors regulating fission (e.g., dynamin-related protein 1 (Drp1) and mitochondrial fission 1 protein (Fis1)) or fusion (e.g., mitofusin 2 (MFN2), and optic atrophia 1 (OPA1)) [26]. Exposure to HG increased protein levels of the fission protein Drp1 in HUVECs. In contrast, other components of the mitochondrial dynamic machinery remained essentially unchanged following HG insults (Fig. 2A–E). Administration of MSCs markedly decreased the expression of Drp1. Notably, none of these beneficial effects mediated by the MSCs were mimicked when cocultured with HSFs. In addition, we also examined the expression of these mitochondrial dynamics factors in vivo models of diabetic mice. Analogously, MSCs infusion reduced Drp1 expression in *db/db* mice but had no effect on the expression of Fis1, MFN2, and OPA1 in mouse aortas (Fig. 2F–J), implying that Drp1 is a critical target of MSCs-mediated mitochondrial dynamics improvement.

Because Drp1 is a key regulator of mitochondrial fission, which can self-assemble into large multimeric spirals, mediating mitochondrial fission via its GTPase activity, we next examined whether MSCs maintain mitochondrial dynamics via regulating Drp1 activity. We found that HUVECs exposed to HG exhibited a significant increase in Drp1-GTPase activity, whereas cocultured with MSCs markedly blunted HG-induced Drp1 activity in HUVECs (Fig. 2K). Since Drp1 translocation to the mitochondria is a key event in mitochondrial fission, we thus determined Drp1 distribution by subcellular fractionation analysis. As shown in Fig. 2L, HG insults induced an accumulation of Drp1 in the mitochondrial fraction compared with that of control group, whereas treatment with MSCs impeded Drp1 recruitment to the mitochondria. In addition, confocal microscopy further corroborated the fused and branched mitochondrial phenotype seen in MSC-treated cells relative to HG group alone (Fig. 2M). What is more, HUVECs transfected with a dominant-negative mutant form of Drp1 (Drp1-K38A) rescued HG-induced mitochondrial fragmentation, which further indicated that Drp1-mediated mitochondrial fission is indeed involved in mitochondrial fragmentation upon HG insults. Taken together, these observations suggest that MSCs might regulate mitochondrial dynamics by blocking Drp1 expression and function.

### STC1 is critical for MSCs to inhibit Drp1-mediated mitochondrial fission and improve mitochondrial fitness

**Fig. 3:**
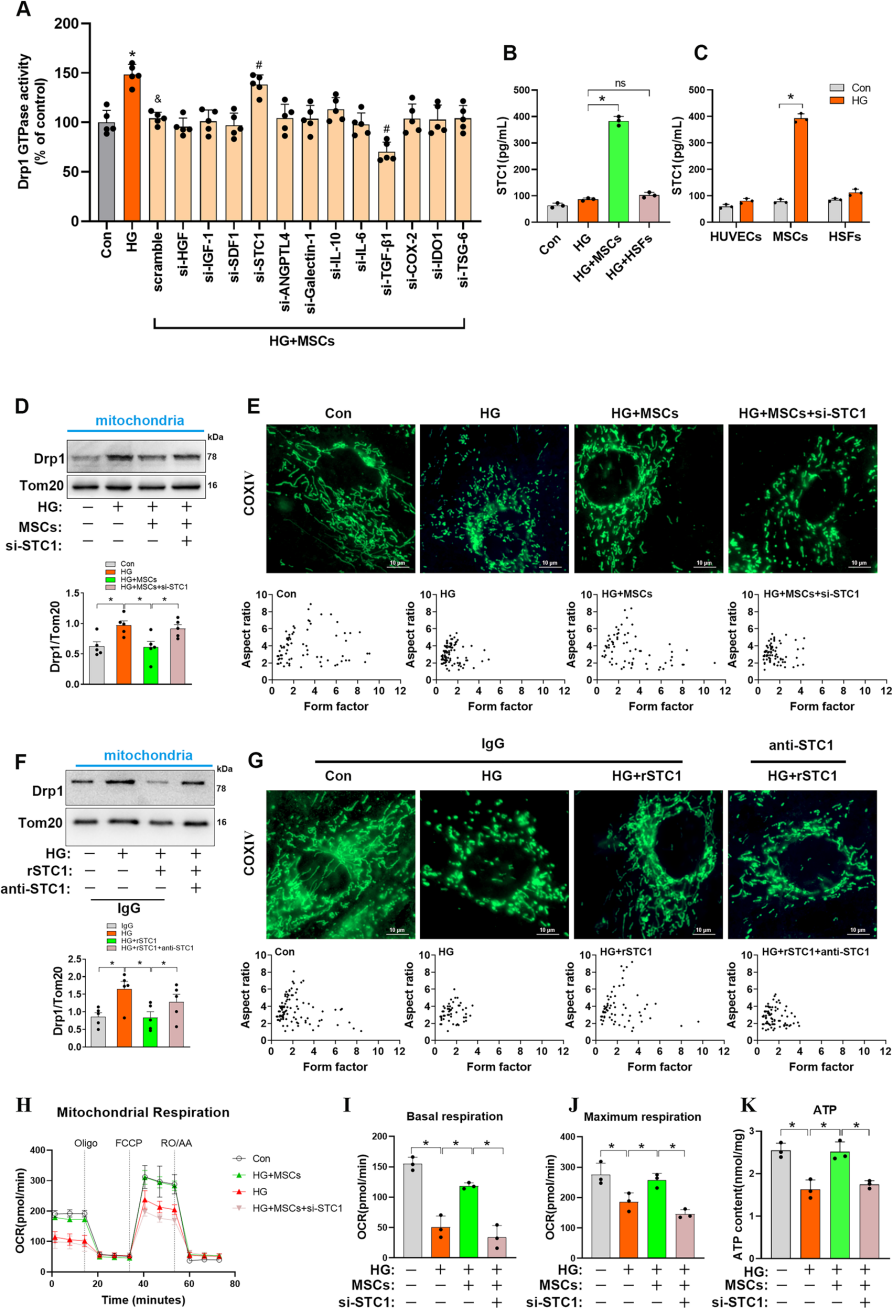
MSC-derived STC1 counteracts HG-induced mitochondrial fission and mitochondrial compromise in a Drp1-dependent manner. **A** HUVECs were transfected with a scramble siRNA or siRNAs directed against the candidate secretory molecules, lysates immunoprecipitated with anti-Drp1 antibody were used to measure Drp1 GTPase activity (*n* = 5 independent experiments). Data are shown as means ± SD. (*P < 0.05 vs. Conn, ^*&*^*P* < 0.05 vs^.^ HG, ^*#*^*P* < 0.05 vs. HG+MSCs^*+*^scramble). **B** STC1 levels in culture media of HUVECs from different groups were detected by ELISA (*n* = 3 independent experiments). Data are shown as means ± SD. ns indicates not significant. *P < 0.05. **C** STC1 levels in culture media of HUVECs, MSCs, and HSFs in the presence or absence of HG incubation were quantified by ELISA (*n* = 3 independent experiments). Data are show*n* as means ± SD. *P < 0.05. **D** Representative Western blots and densitometry analysis of mitochondrial Drp1 in HUVECs upon HG insults when cocultured with MSCs transfected with scramble siRNA or with STC1 siRNA (*n* = 5 independent experiments). Data are shown as means ± SD. *P < 0.05. **E** Micrographs of mitochondrial morphology were visualized by anti-COXIV antibody staining in HUVECs cocultured with MSCs transfected with scramble siRNA or with STC1 siRNA (*n* = 60–100 cells/group). **F** Representative Western blots and densitometry analysis of mitochondrial Drp1 in HUVECs treated with rSTC1 (50 ng/mL), anti-rSTC1 antibody or control IgG (*n* = 5 independent experiments). Data are shown as means ± SD. *P < 0.05. **G** Micrographs of mitochondrial morphology were visualized by anti-COXIV antibody staining in HUVECs treated with rSTC1, anti-rSTC1 antibody or control IgG (*n* = 60–100 cells/group). **H**–**J** Basal and maximal mitochondrial oxygen consumption rates (OCRs) in HUVECs were determined by Seahorse XF96 analyses (*n* = 3 technical replicates). **K** ATP levels were quantified in HUVECs (*n* = 3 i*n*dependent experiments). Data are shown as means ± SD. *P < 0.05. Stude*n*t’s *t* test (unpaired) for the comparisons of two groups, and one-way ANOVA with Tukey’s post hoc test if more than two groups were compared.

To search for MSCs-derived factors that play a role in regulating mitochondrial dynamics remodeling under HG conditions, we screened a panel of candidate molecules secreted from MSCs that have previously been reported to be involved in the regulation of inflammation, immune responses, and oxidative stress [16, 27–29]. To confirm the role of the candidate molecules, each gene was knocked down in MSCs with the gene-specific small interfering RNA (siRNA) transfection (Fig. S1A–L), and the effects of MSCs with the gene knockdown on Drp1-GTPase activity were tested. Results showed that MSCs with STC1 knockdown were less effective at suppression of Drp1 activity in HG conditions than MSCs with scramble siRNA (Fig. 3A); however, the knockdown of other secretory-protein-encoding genes in MSCs did not reverse the efficacy of MSCs on Drp1 activity, except TGF-β1, whose depletion seems to give rise to a more significant inhibition on Drp1 activity. We further confirmed the increased levels of STC1 in the supernatant of HUVECs from MSCs-cocultured groups compared with HG groups alone, whereas no major alteration was observed when HUVECs cocultured with HSFs (Fig. 3B). To ascertain the potential sources of STC1, HUVECs, MSCs, and HSFs were cultured separately under normal or HG conditions, and the STC1 levels were determined in supernatant. As expected, there was a marked increase in STC1 secretion from MSCs after exposure to HG, whereas no obvious increase in levels of secreted STC1 was present in supernatant of HUVECs or HSFs (Fig. 3C), suggesting that STC1 secretion was specific for MSCs. Together, these observations suggest that MSCs were activated by HG to upregulate and secrete increased amounts of STC1 that inhibited Drp1 activity.

Next, to test whether STC1 secreted by MSCs was involved in inhibiting mitochondrial fission in HUVECs under HG conditions, MSCs were transfected with siRNA for STC1 (Fig. S2A, B), and Drp1 translocation to the mitochondria was determined by cell fractionation experiments. We first validated the secretion of STC1 in co-culture systems. As indicated in Fig. S2C, si-STC1 MSCs produced less STC1 in the cell culture supernatant. Furthermore, MSCs administration decreased Drp1 protein levels in the mitochondrial fractions of HUVECs compared with HG group alone, whereas knockdown of STC1 in MSCs negated this phenotype (Fig. 3D). Accordingly, these observations were also confirmed by immunofluorescence assay, as MSCs transfected with si-STC1 were less effective than control MSCs in suppressing HG-induced mitochondrial fragmentation (Fig. 3E). In addition, to further evaluate the role of STC1 in mitochondrial dynamics remodeling, we supplemented recombinant STC1 (rSTC1) in the culture medium of HUVECs in response to HG insults. In agreement with the aforementioned observation, rSTC1 supplementation prevented Drp1 translocation to the mitochondria and maintained elongated mitochondrial morphology of HUVECs in HG conditions (Fig. 3F, G); however, these favorable effects were blocked by administration of STC1-neutralizing antibody but not by an isotype control of IgG, indicating that MSC-derived STC1 is sufficient to counteract HG-induced mitochondrial fission in a Drp1-dependent manner.

To further understand the functional consequences of STC1 in preventing Drp1-mediated mitochondrial fission in HUVECs, we therefore examined the effects of STC1 on mitochondrial fitness. Indeed, si-STC1 transfection was sufficient to abrogate the improvement of basal and maximal rates of oxygen consumption conferred by MSCs coculture, and this paralleled a marked decrease in mitochondrial ATP generation (Fig. 3H–K).

### ERK1/2 inhibition by STC1 restrains Drp1 recruitment to mitochondria

**Fig. 4:**
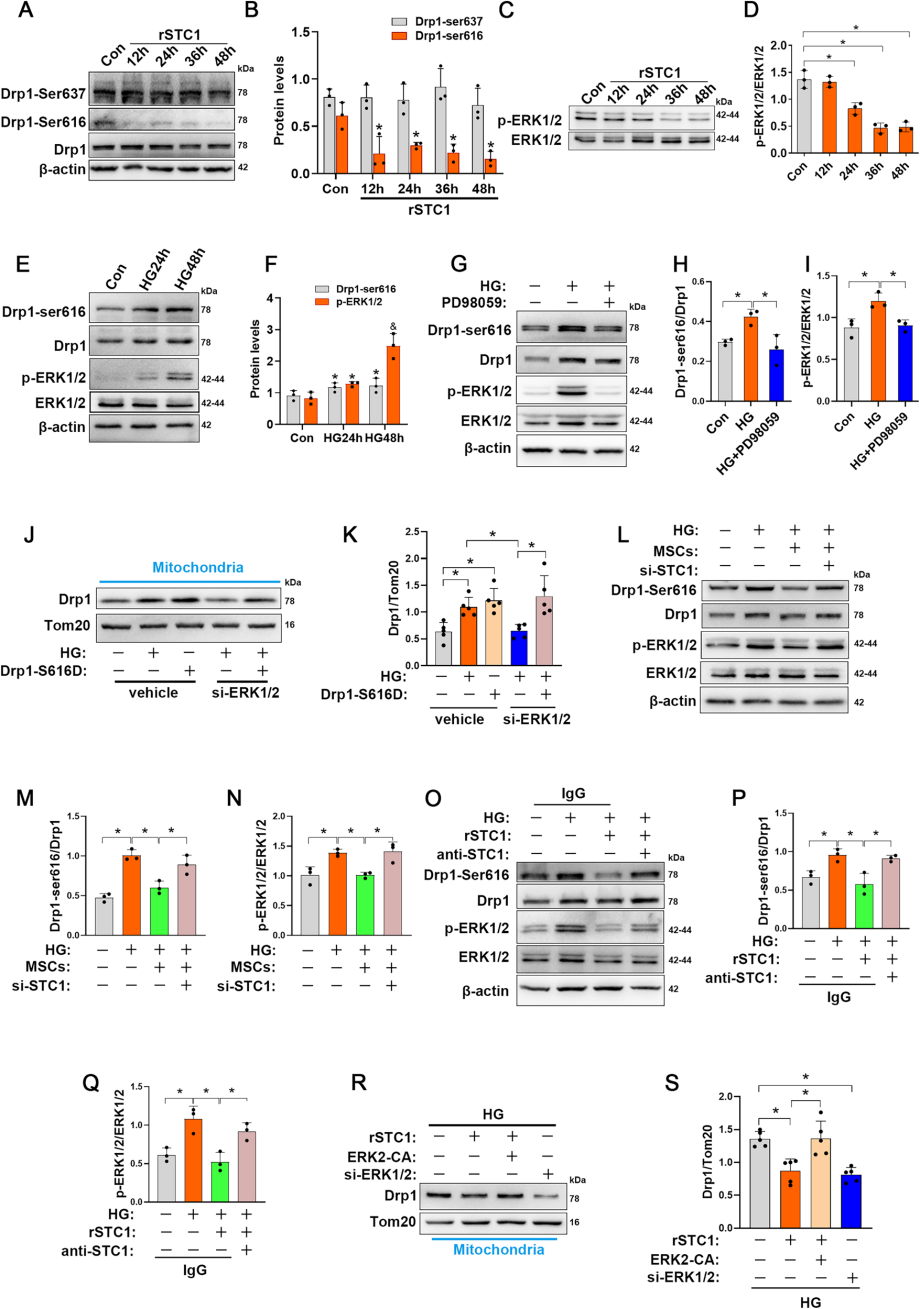
STC1 regulates mitochondrial dynamics remodeling through ERK1/2-Drp1 axis. **A**, **B** HUVECs were treated with rSTC1 for indicated times, and Drp1-Ser637 and Drp1-Ser616 protein levels were detected by Western blot (*n* = 3 independent experiments). Data are shown as means ± SD. *P < 0.05. **C**, **D** HUVECs were treated with rSTC1 for indicated times, and p-ERK1/2 protein levels were detected by Western blot (*n* = 3 indepe*n*dent experiments). Data are shown as means ± SD. *P < 0.05. **E**, **F** HUVECs were incubated with HG for 24 h or 48 h, and lysates were Western blotted for indicated proteins (*n* = 3 independent experiments). Data are shown as means ± SD. (*P < 0.05 vs. Conn, ^*&*^*P* < 0.05 vs. HG24h) **G**–**I** HUVECs were incubated with HG in the presence or absence of PD98059 (10 μM) for 48 h, and lysates were Western blotted for indicated proteins (*n* = 3 independent experiments). Data are show*n* as means ± SD. *P < 0.05. **J**, **K** Representative Western blots and bar graphs of Drp1 of mitochondrial fractions obtained from HUVECs in each group (*n* = 5 independent experiments). Data are shown as means ± SD. *P < 0.05. **L**–**N** MSCs transfected with scrambled or STC1-specific siRNA were co-cultured with HUVECs in medium containing HG, representative Western blots and densitometry analysis of p-Drp1 and p-ERK1/2 levels in each group (*n* = 3 independent experiments). Data are shown as means ± SD. *P < 0.05. **O**–**Q**, HUVECs were exposed to HG with or without rSTC1, control IgG or anti-STC1 antibodies were added to the HUVECs, and lysates were Western blotted for indicated proteins (*n* = 3 independent experiments). **R**, **S** Representative Western blots and bar graphs of Drp1 in mitochondrial fractions obtained from HUVECs in each group (*n* = 5 independent experiments). Data are shown as means ± SD. *P < 0.05. Data were analyzed using one-way ANOVA with Tukey’s post hoc test.

Because very little was known about the effect of STC1 on regulation of subcellular localization of Drp1, we aimed to elucidate the regulatory mechanisms by which STC1 inhibited activity of Drp1. Mitochondrial fission is regulated through recruitment of Drp1 to mitochondrial membranes, during which the fission activity of Drp1 is closely associated with its phosphorylation status at different amino acid sites, including phosphorylation of Ser616, which promotes Drp1 activity, and Ser637, which inhibits its activity [30]. We initially questioned whether STC1 can modify the phosphorylation status of Drp1. Indeed, we observed a time course-dependent decrease in Ser616 phosphorylation upon rSTC1 treatment, but not at Ser637 (Fig. 4A, B). Since STC1 has no known kinase activity, we speculated that it may indirectly regulate Drp1 phosphorylation levels. To address if kinase(s) are intermediate effector(s) of the STC1-Drp1 pathway, we performed an analysis of the sequences surrounding Ser616. Remarkably, it represents a perfect consensus sequence for phosphorylation by ERK1/2, and is highly conserved among species (Fig. S2D). Given that ERK1/2 has previously been demonstrated to drive mitochondrial fission by promoting phosphorylation of Ser616 and recruitment Drp1 to the mitochondrial outer membrane [31], we thus speculate this kinase may participate STC1-induced mitochondrial dynamics remodeling in endothelial cells. Notably, rSTC1 treatment induced a time course-dependent inhibition on phosphorylation of ERK1/2, implying an inhibition of ERK1/2 signaling (Fig. 4C, D).

It is known that ERK1/2 is usually activated under HG conditions [32, 33], we thereby evaluated whether ERK1/2 could directly regulate Drp1 in mouse aortas under diabetic conditions. Immunoprecipitation analyses demonstrated that interaction between ERK1/2 and Drp1 was robustly increased in aortas tissues of *db/db* mice (Fig. S2E). In the current study, we also observed that HG induced a prominent hyperphosphorylation of ERK1/2 in HUVECs, which is closely followed by an increase in Ser616-phosphorylated Drp1 (Fig. 4E, F). In addition, when incubated HUVECs with HG in the presence of the MEK inhibitor PD98059 to inhibit ERK1/2 signaling, a marked decrease in Drp1-Ser616 phosphorylation was observed, which closely tracked the inhibition of ERK1/2 phosphorylation (Fig. 4G–I). Furthermore, to determine the specific effect of Drp1-Ser616 on mitochondria pathology, cells were transfected with Drp1-S616D (constitutively active form of Drp1) and evaluated for mitochondrial morphology and function under diabetic conditions. Ectopic expression of Drp1-S616D induced even a more elevated level of Drp1 protein in mitochondria compared with HG group alone; ERK1/2-silencing treatment largely blocked HG-induced Drp1 translocation to mitochondria, whereas introduction of Drp1-S616D negated the effect of ERK1/2 knockdown on Drp1 translocation (Fig. 4J, K and S2G). These findings were further confirmed by immunofluorescence staining showing that ERK1/2 knockdown with specific siRNA reversed HG-induced mitochondrial fragmentation (Fig. S3A, B); however, when Drp1-S616D was introduced simultaneously, this phenotype was abolished, suggesting that the interaction between ERK1/2 and Drp1 mediates the alteration of mitochondrial morphology under diabetic condition. Importantly, simultaneous analysis of mitochondrial oxygen consumption rate (OCR) by Seahorse analyze indicated that genetic inhibition of ERK1/2 was sufficient to improve basal and maximal mitochondrial OCRs upon HG insults, and this was accompanied by a marked decrease in mtROS, as well as the restoration of mitochondrial membrane potential (Ψ_m_) and ATP generation (Fig. S3C–J); however, the favorable phenotypes were eliminated upon introduction of Drp1-S616D, suggesting that the phosphorylation of serine 616 is an essential component to HG-induced mitochondrial fission and mitochondrial compromise.

Finally, to confirm whether MSCs-secreted STC1 inhibits mitochondrial fission by suppressing HG-induced constant activation of ERK1/2 signaling, we next examined the phosphorylation status of both Drp1 and ERK1/2 in HUVECs. MSCs treatment decreased HG-induced ERK1/2 phosphorylation in parallel with a decline in Drp1-Ser616 phosphorylation, whereas this phenotype was abolished when STC1 was knocked down in MSCs (Fig. 4L–N). In addition, rSTC1 supplementation could also mimic the regulatory effect of MSCs on ERK1/2-Drp1 signaling (Fig. 4O–Q), however, these results were failed to be replicated when STC1-neutralizing antibody was administrated into the culture medium. Furthermore, either supplementation of rSTC1 or genetic inhibition of ERK1/2 blocked the translocation of Drp1 to mitochondria induced by HG and preserved interconnected mitochondrial network (Figs. 4R, S and S4A, B); however, ectopic expression of ERK2 (Fig. S2F) prevented the inhibitory effect mediated by rSTC1 supplementation on Drp1 translocation and alterations in mitochondrial morphology, demonstrating that STC1 indeed functions upstream of ERK1/2. Accordingly, either supplementation of rSTC1 or genetic inhibition of ERK1/2 improved HG-induced mitochondrial compromise, as displayed by increased basal and maximal OCRs, along with decrease of mtROS, increase of Ψ_m_ and ATP generation (Fig. S4C–J); however, the ectopic expression of ERK2 was sufficient to hinder the improvement on mitochondrial function mediated by rSTC1 supplementation. Collectively, these data suggested that MSCs-derived STC1 regulates mitochondrial dynamics remodeling through ERK1/2-Drp1 axis and, consequently, improve mitochondrial metabolism and function.

### MSCs suppress HG-induced endothelial inflammation and apoptosis by STC1-dependent mitochondrial dynamics remodeling

**Fig. 5:**
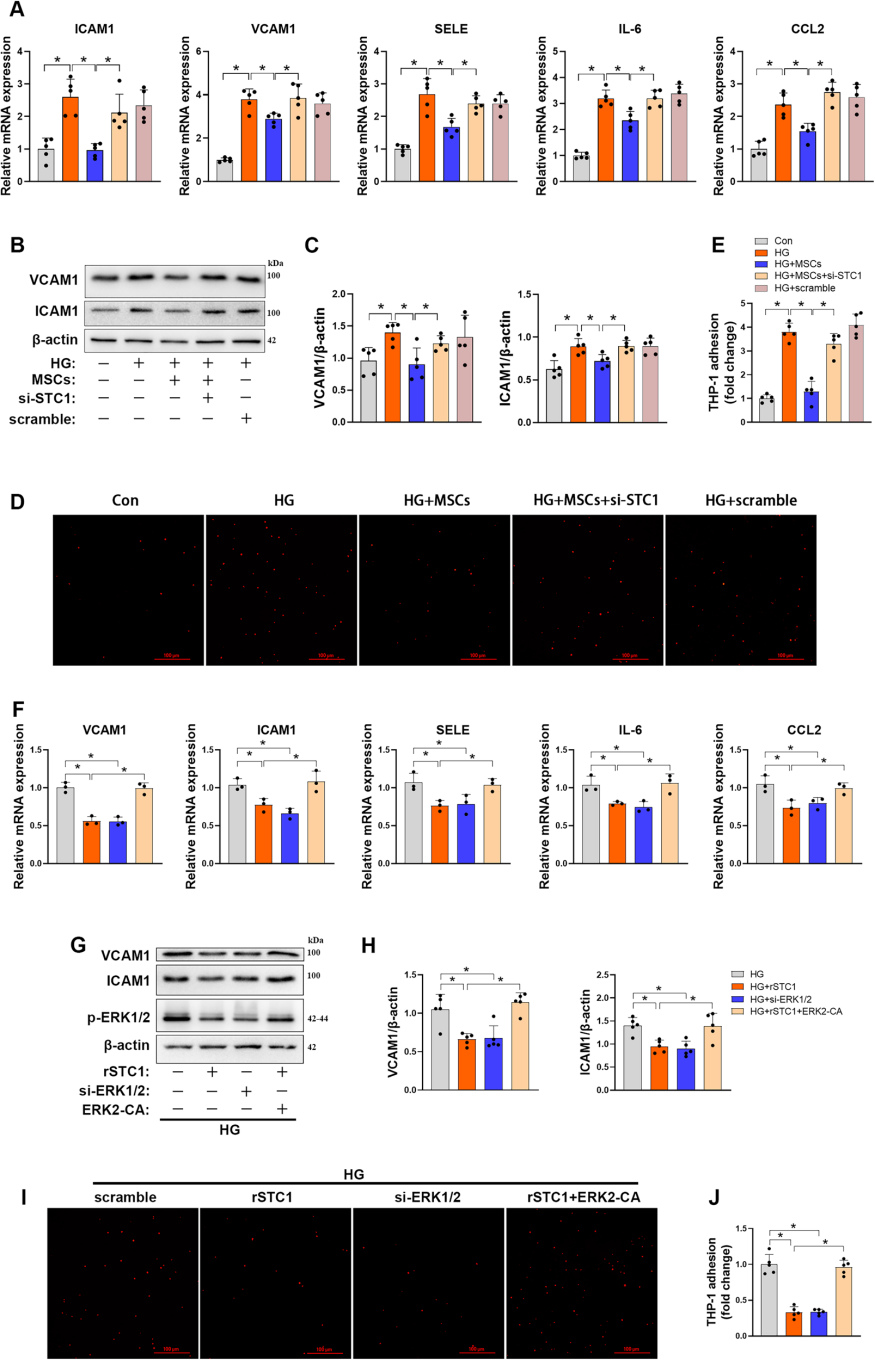
MSCs suppress HG-induced endothelial inflammation by STC1-dependent mitochondrial dynamics remodeling. **A** HUVECs were cocultured with MSCs transfected with scramble siRNA or with STC1 siRNA, mRNA expression of inflammatory adhesion molecules was analyzed by qPCR (*n* = 5 independent experiments). Data are shown as means ± SD. *P < 0.05. **B**, **C** Representative Western blots and densitometric analysis of protein levels of ICAM1 and VCAM1 in HUVECs from each group (*n* = 5 indepe*n*dent experiments). Data are shown as means ± SD. *P < 0.05. **D**, **E** Representative images and corresponding quantitation showed the adhesion of monocytes to the lawn of HUVECs (*n* = 5 independent experiments). Data are shown as means ± SD. *P < 0.05. **F** HUVECs were exposed to HG, and transfected with si-ERK1/2 or treated with rSTC1 and/or transfected with constitutively active ERK2 (ERK2-CA), mRNA expression of inflammatory adhesion molecules was analyzed by qPCR (*n* = 3 independent experiments). Data are shown as means ± SD. *P < 0.05. **G**, **H** Cells lysates were analyzed by Western blot analysis for the expression of ICAM1 and VCAM1, as illustrated (*n* = 5 independent experiments). Data are show*n* as means ± SD. *P < 0.05. **I**, **J** Representative images and corresponding quantitation show the adhesion of monocytes to the lawn of HUVECs (*n* = 5 independent experiments). Data are shown as means ± SD. *P < 0.05. Data were analyzed using one-way ANOVA with Tukey’s post hoc test.

Endothelial dysfunction is characterized by endothelial inflammation and oxidative stress, which are associated with alteration of mitochondrial dynamics [3, 34]. Having established a strong correlation between the effect of MSCs on mitochondrial dynamics remodeling and secretion of STC1, we next investigated the functional link between STC1-dependent mitochondrial dynamics remodeling and endothelial injury. Results from qPCR and immunoblotting assays revealed that coculture with MSCs attenuated HG-induced mRNA or protein upregulation of the proinflammatory adhesion molecules VCAM1, ICAM1, and SELE, chemokine CCL2 and cytokine IL6, which accompanied with decreased monocyte adhesion to inflamed HUVECs (Fig. 5A–E); however, such phenotypes were blocked when STC1 was knocked down with siRNA. Furthermore, overexpression of dominant-negative Drp1 (DN-Drp1) or pharmacological inhibition by Mdivi-1, a specific Drp1 inhibitor, prevented STC1 knockdown-induced mtROS elevation, which paralleled a marked decrease in mRNA expression of vascular proinflammatory genes, and subsequently monocyte adhesion (Fig. S5A–E), indicating that MSCs suppress HG-induced inflammatory response, in part, by STC1-mediated inhibition of Drp1 activity. To further investigate whether the manipulation of mitochondrial dynamics through the ERK1/2-Drp1 axis is responsible for the anti-inflammatory effect of STC1, we utilized a genetic approach to assess the role of the ERK1/2-Drp1 axis. rSTC1 supplementation blunted HG-induced increase in mRNA or protein expression of the proinflammatory adhesion molecules, in parallel with decreased monocyte adhesion. Such phenotype was recapitulated by genetic inhibition of ERK1/2 (Fig. 5F–J). However, the beneficial effects of rSTC1 supplementation disappeared when ERK2 was simultaneously overexpressed. Additionally, neither ERK1/2 knockdown nor rSTC1 supplementation could prevent HG-induced proinflammatory phenotype when Drp1-S616D was introduced (Fig. S5F–H). These observations demonstrate that STC1-ERK1/2-Drp1 axis-mediated mitochondrial dynamics remodeling is essential for MSCs to combat against HG-induced endothelial inflammation.

Disturbed mitochondrial dynamics is associated with cell death, since mitochondrial dynamics-mediated cristae remodeling has been recognized as a crucial contributor to cytochrome C mobilization and the activation of the apoptotic pathway [35]. Thus, we next studied the effect of STC1-ERK1/2-Drp1 signaling on cellular apoptosis in response to HG. As shown in Fig. S6A, activated Bax translocated from the cytoplasm to the mitochondria in response to HG, which was accompanied by cytochrome C release to the cytosol. However, MSCs treatment reversed the Bax and cytochrome C redistribution, indicating that the protective effect achieved by MSCs treatment may be involved in the prevention of the mitochondrial apoptotic pathway. Next, we investigate whether STC1 participates in the MSCs-mediated antiapoptotic process. Mitochondrial fractionation experiments indicated STC1 knockdown halted the effect of MSCs against HG-induced redistribution of Bax and cytochrome C (Fig. S6B–D). Parallel to this, MSCs treatment inhibited HG-induced increase in expression of C-caspase-3, whereas STC1 knockdown abrogated the anti-apoptotic effect of MSCs (Fig. S6E, F). Consistent results were also observed when analyzed with flow cytometry (Fig. S6G, H). Furthermore, consistent with the observation of endothelial inflammation, ectopic expression of ERK2 damped the anti-apoptotic effect of rSTC1 supplementation in HG circumstances (Fig. S6I, J). Genetic inhibition of ERK1/2 also protected against HG-induced apoptosis; however, this beneficial effect was blunted when Drp1-S616D was introduced. Collectively, these results suggest that the protective effect of MSCs against HG-induced endothelial inflammation and apoptosis is mediated by STC1-ERK1/2-Drp1-dependent remodeling of mitochondrial dynamics.

### Intravenous administration of MSCs attenuates mitochondrial fission and improves mitochondrial fitness in diabetic mice

**Fig. 6:**
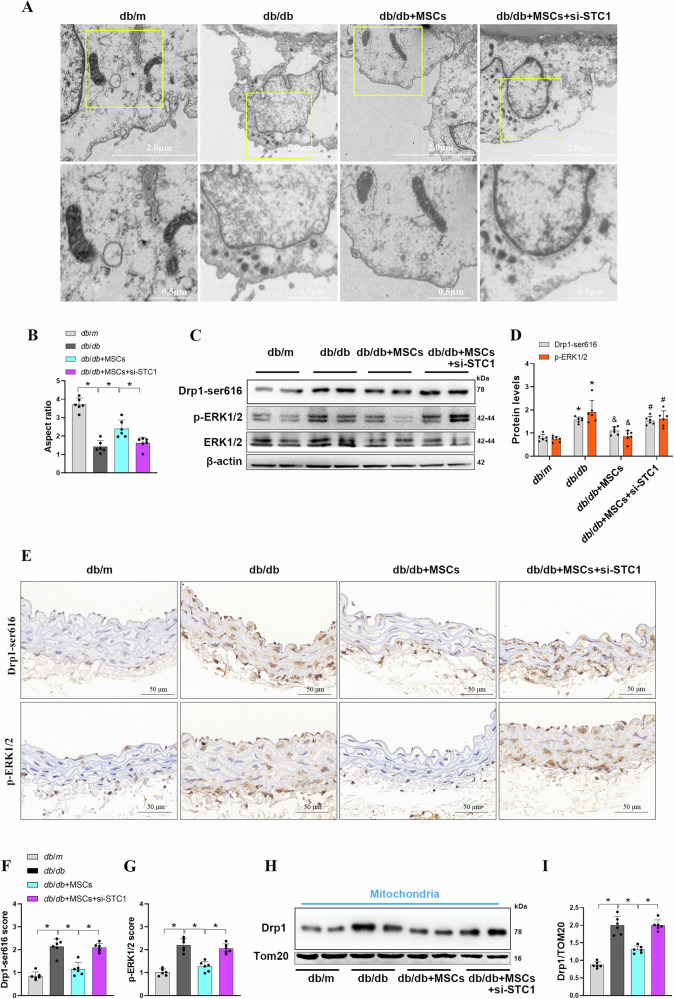
Intravenous administration of MSCs attenuates mitochondrial fission in diabetic mice. **A** Representative TEM micrographs of mitochondria in aortic endothelial cells from mice of different groups. **B** Quantification of mitochondrial aspect ratio in aortic endothelial cells from mouse (*n* = 6 mice/group). Data are shown as means ± SD. *P < 0.05. **C**, **D** Representative Western blots and densitometric analysis of indicated proteins expression in aorta of *db*/*m* and *db*/*db m*ice given saline or MSCs transfected with scramble siRNA or with STC1 siRNA (*n* = 6 mice/group). Data are shown as means ± SD. (*P < 0.05 vs. *db*/*m*, ^*&*^*P* < 0.05 vs. *db*/*db*, ^*#*^*P* < 0.05 vs. *db*/*db* + MSCs). **E**–**G** Representative images of immunohistochemical staining and quantification of positive staining for Drp1-ser616 and p-ERK1/2 staining in sections of aorta from different groups (*n* = 6 mice/group). Data are shown as means ± SD. *P < 0.05. **H**, **I** Representative Western blots and graph of Drp1 of mitochondrial fractions obtained from aorta tissues in each group (*n* = 6 mice/group). Data are shown as means ± SD. *P < 0.05. Data were analyzed using one-way ANOVA with Tukey’s *post hoc* test.

To explore the relevance of our in vitro findings, we determined the effect of MSCs therapy in *db/db* mice. As shown in Fig. S7A, the serum level of STC1 in *db/db* mice treated with MSCs was significantly higher compared to that in the *db/db* alone; however, knockdown of STC1 in MSCs negated the upregulation of STC1 in mouse serum. Analysis of mitochondrial ultrastructure in endothelial cells of aorta from *db/db* mice revealed more dense and fragmented mitochondria compared with *db/m* controls, intravenous administration of MSCs markedly prevent mitochondrial fission in *db/db* mice; whereas the morphologic improvement was hindered in groups with si-STC1-transfected MSCs (Fig. 6A). We quantified these changes as a measure of AR and found that mitochondria from MSCs-treated mice phenocopied those from nondiabetic controls in a STC1-dependent manner (Fig. 6B). In addition, immunoblotting assay revealed that MSCs infusion blocked hyperphosphorylation of ERK1/2 in aorta from *db/db* mice, which accompanied with decreased Drp1-Ser616 phosphorylation; however, STC1 knockdown abrogated this phenomenon (Fig. 6C, D). These findings were further corroborated by immunohistochemical staining (Fig. 6E–G). Consistently, cell fractionation experiments also confirmed that depletion of STC1 nullified the effect of MSCs on preventing Drp1 translocation to the mitochondria (Fig. 6H, I), indicating that STC1 is essential for MSCs to prevent impairment of mitochondrial morphology in diabetic mice by inhibiting ERK1/2-mediated Drp1 activation.

Since alteration of mitochondrial architecture was associated with degeneration of mitochondrial fitness [11, 36]. We next evaluated the impact of MSCs infusion on mitochondrial status in vivo. Functional mitochondrial pool was analyzed by co-staining with Mitotracker Green and Deep Red, dyes that are taken up in a ΔΨ_m_-independent and -dependent manner, respectively. We observed an increase in dysfunctional mitochondria (MitoTracker Green^+high^, MitoTracker Red^+low^) of isolated aortic mitochondria from *db/db* mice, and this paralleled a marked decrease in ATP (Fig. S7B, C). MSCs infusion prevented the accumulation of dysfunctional mitochondria with loss of ΔΨ_m_ and enhanced ATP generation, whereas si-STC1-transfected MSCs had minimal effect on reversing these phenotypes. These results suggest that MSCs treatment improves mitochondrial fitness in the diabetic milieu through the secretion of STC1.

### Intravenous administration of MSCs mitigates vascular dysfunction and inflammation in diabetic mice

**Fig. 7:**
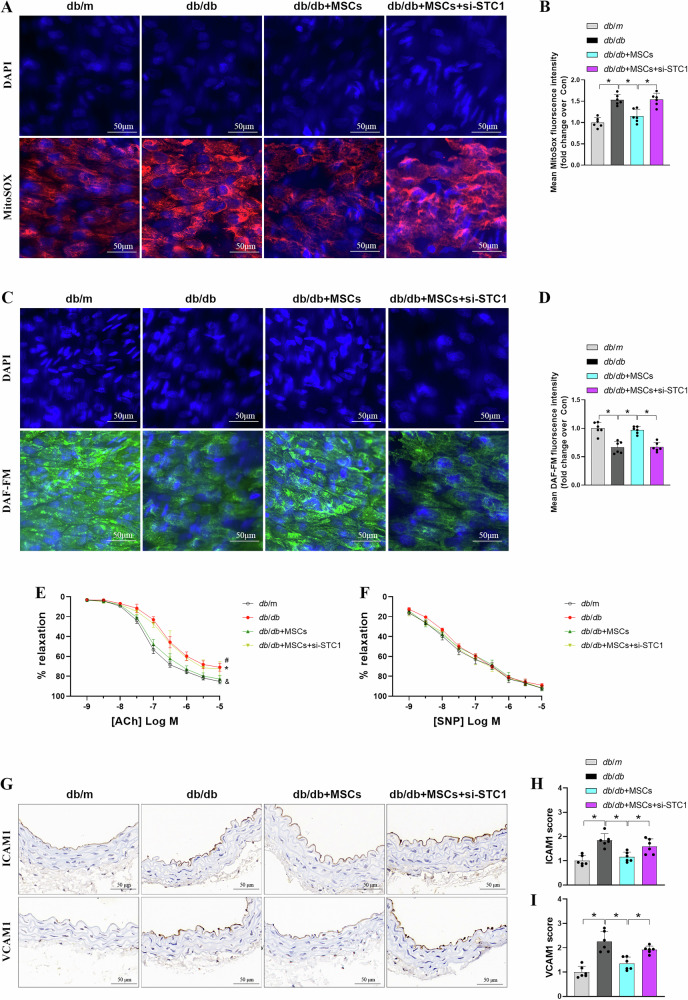
Intravenous administration of MSCs mitigates vascular dysfunction in diabetic mice. **A**, **B** Aortas were isolated and prepared en face from *db*/*m* and *db*/*db* mice and stained for MitoSOX, fluorescence was calculated as mean fluorescence intensity (MFI) (*n* = 6 mice/group). Data are shown as means ± SD. *P < 0.05. **C**, **D** DAF-FM staining was performed to assess NO, quantification of NO levels was based on measurement of MFI (*n* = 6 mice/group). Data are shown as means ± SD. *P < 0.05. **E**, **F** Acetylcholine (Ach)-induced endothelium-dependent and sodium nitroprusside (SNP)-induced endothelium-independent vasorelaxation in *db*/*m* and *db*/*db* mice aorta precontracted with phenylephrine (*n* = 6 mice/group). Data are shown as means ± SD. (*P < 0.05 vs. *db*/*m*, ^*&*^*P* < 0.05 vs. *db*/*db*, ^*#*^*P* < 0.05 vs^.^ *db*/*db* + MSCs). **G**–**I** Representative images of immunohistochemical staining and quantification of positive staining for ICAM1 and VCAM1 staining in sections of aorta from different groups (*n* = 6 mice/group). Data are shown as means ± SD. *P < 0.05. Data were analyzed using one-way ANOVA with Tukey’s post hoc test.

We next asked if MSCs infusion could combat hyperglycemia-induced oxidative stress in vivo, which is inextricably linked to excessive mitochondrial fission and mitochondrial dysfunction in a diabetic environment [37]. En face endothelium MitoSOX staining showing that MSCs treatment blunted the elevated mtROS level in endothelium of mouse aortas from *db/db* mice, while knockdown of STC1 abrogated this manifestation (Fig. 7A, B). Formation of NO by endothelial nitric oxide synthase (eNOS) is a central process in the homeostatic regulation of vascular functions [38]. Given the association between oxidative stress and perturbations in NO bioavailability in human and animal models of cardiovascular diseases [4], we determined NO production in mouse aortas. Here, we observed MSCs treatment reversed the decline of NO production in aortas from *db/db* mice (Fig. 7C, D), whereas transfection of MSCs with the si-STC1 resulted in nullification of this improvement. Accordingly, these observations were further confirmed by the immunoblotting analysis of phosphorylation of eNOS at serine 1177 both in vitro and in vivo (Fig. S8A, B).

Since NO is a key determinant of vascular health that promotes vascular relaxation and modulates vascular inflammation and leukocyte adhesion [39], we next examined the effect of MSCs infusion in endothelium-dependent vasorelaxation. Figure 7E, F shows that MSCs injection restored acetylcholine (ACh)-mediated vasodilation without affecting endothelium-independent sodium nitroprusside (SNP)-induced vasodilation in aortic rings from *db/db* mice; however, such effect on ACh-mediated vasodilation was abolished when STC1 was knocked down in MSCs. We further determined whether inhibition of ERK1/2-Drp1 signaling-mediated mitochondrial fission participate in STC1-induced improvement of endothelial function. Segments of arteries from *db*/*db* mice were studied with preincubations of either Mdivi-1 or PD98059 ex vivo after infusion of si-STC1-transfected MSCs. Pharmacological inhibition of either Drp1 or ERK1/2 signaling reversed the impairment of ACh-induced relaxation in *db/db* mice treated with MSCs caused by STC1 knockdown (Fig. S8C, D), whereas there was no significant difference in endothelium-independent relaxation among these groups, indicating that MSCs injection prevent endothelial dysfunction in diabetic mice through STC1-dependent inhibition of mitochondrial fission.

Lastly, we tested whether administration of MSCs could regulate endothelial inflammation in diabetic mice via manipulating mitochondrial dynamics remodeling. Immunohistochemical assay revealed that the expression of ICAM1 and VCAM1 was higher in the aortic endothelium from *db/db* mice than *db/m* control mice (Fig. 7G–I). MSCs treatment attenuated the expression of ICAM1 and VCAM1 in the aortic endothelium, which was enhanced by diabetes；however, the anti-inflammatory phenotype of MSCs was abolished when STC1 was knocked down. Additionally, qPCR analysis of mRNA expression of proinflammatory genes also confirmed identical results (Fig. S8E). In conclusion, MSCs potentially exert vascular protection effect through STC1-ERK1/2-Drp1 axis-dependent mitochondrial dynamics remodeling.

## Discussion

In the current study, we uncover a novel mechanism by which MSCs therapy inhibit hyperglycemia-induced endothelial dysfunction in diabetes mellitus. Critically, STC1 was identified as a crucial regulator secreted from MSCs, which preserves mitochondrial morphology and functional integrity through inhibiting ERK1/2-Drp1 signaling-mediated mitochondrial fission, thus preventing hyperglycemia-induced oxidative injury, endothelial inflammation and mitochondrial apoptotic cascade, and thereby protecting against endothelial dysfunction. These findings established a strong causal link between the protective paracrine effect of MSCs and the maintenance of mitochondrial dynamic homeostasis in hyperglycemia-injured endothelial cells in diabetic states.

Accumulating evidence suggests that mitochondrial dysfunction and oxidative stress contribute critically to the pathogenesis of endothelial dysfunction in patients with diabetes mellitus [3, 40]. In the endothelial cells, mitochondria play a key role in the regulation of various metabolic processes and mainly function as ROS signaling organelles, maintaining cellular redox homeostasis [41]. Mitochondria are dynamic organelles balanced by the flux of iterative fusion and fission events. Altered mitochondrial dynamics have been proved not only contribute to alterations in mitochondrial morphology and function degeneration, but also facilitate ROS production and oxidative stress in diabetes mellitus [41]. For example, Yu et al. reported that excessive fission-induced mitochondrial fragmentation is a causal factor necessary for HG-induced ROS elevation [10]. In addition, PDIA1 depletion induces vascular diseases associated with diabetes or aging through promoting Drp1-mediated mitochondrial fragmentation and ROS overproduction [11]. Consistently, we provide compelling evidence showing that increased Drp1 protein levels and mitochondrial fragmentations occurred in HG-treated HUVECs and aortas of diabetic mice. Of note, the expression patterns of other dynamic proteins such as Fis1, OPA1, and MFN2 were not significantly affected. In line with our findings, Drp1 expression was also reported to be specifically induced in aortic endothelial cells during diabetes-accelerated atherosclerosis and in diabetic *db/db* hippocampus [4, 42]. As anticipated, administration of MSCs suppressed the expression of Drp1 and its GTPase activity, leading to an improvement in the mitochondrial network along with a decrease in mtROS production. Thus, these results support the notion that Drp1 is an important target for MSCs therapy against HG-induced mitochondrial fragmentation and oxidative stress uniquely in endothelial cells.

A number of studies have demonstrated that the therapeutic effects of MSCs are attribute to the soluble paracrine factors. Herein, a screen for soluble molecules whose deficiency blocked the modulatory activity of MSCs against HG-induced Drp1-GTPase activity pointed to STC1 as a critical regulator of MSCs. STC-1, a glycoprotein, is a multifunctional molecule involved in the regulation of oxidative stress, inflammatory responses, and cell apoptosis [43, 44]. Transgenic overexpression of STC1 in mice protects against ischemia-reperfusion kidney injury through suppressing superoxide generation [45]. Consistent results were observed after kidney or endothelial cell-specific knockdown of STC1, which resulted in greater generation of superoxide, cell apoptosis, and kidney failure [46]. In cultured endothelial cells, treatment with STC1 diminishes superoxide generation, the activation of pro-inflammatory signaling pathways, and preserves endothelial barrier function [47]. Data from diabetic nephropathy patients, characterized by microvascular dysfunction, have demonstrated that patients with high levels of STC1 have a better prognosis than patients with low STC1 levels [48]. Using loss- and gain-of-function studies of STC1, we clearly demonstrated that STC1 is critical for MSCs to inhibit Drp1-mediated mitochondrial fragmentation and subsequent mtROS overproduction under HG circumstance, indicating that mitochondrial improvement and inhibition of oxidative stress by MSCs treatment was STC1-dependent.

Transforming growth factor beta (TGF-β1) is a pleiotropic factor that regulates a broad spectrum of biological processes involved in tissue homeostasis and injury response [49]. Studies have shown that MSCs can secrete TGF-β1, which plays a well-documented role in MSC immunomodulation [50, 51]. A substantial body of research has indicated that TGF-β1 may also participate in the regulation of mitochondrial dynamics. In the model of renal fibroblast, TGF-β1 stimulation induced Drp1-depedent mitochondrial fragmentation in kidney fibroblast cells, inhibition of Drp1 activation attenuates TGF-β1-induced mitochondrial fission and fibroblast activation [52, 53]. Consistent results were observed in hepatic stellate cell of liver fibrosis [54]. In the present study, our results demonstrate that MSCs transfected with si-TGF-β1 exert a more pronounced inhibitory effect on Drp1 activity in HUVECs compared to hMSCs with the scrambled siRNA, which provides a novel link between TGF-β1 and mitochondrial dynamics. However, in contrast, TGF-β1 stimulation was reported to increase mitochondrial fusion and reduce mitochondrial fission in vascular progenitor cells [55]. More recently, it has been discovered that TGF-β1 plays a crucial role in inducing mitochondrial fusion and metabolic reprogramming in regulatory T cells through a Smad2/3-dependent mechanism [56]. This discrepancy may be explained by the possibility that the effect of TGF-β1 on mitochondrial morphology is likely tissue-specific and context-dependent. In support of this, TGF-β1 has been reported to induces vessel formation in vivo, intriguingly, TGF-β1 promotes endothelial cell apoptosis in vitro and in vivo [57, 58] but up-regulates endothelial cell expression of VEGF [59], an apparent discrepancy because VEGF protects endothelial cells from apoptosis. Thus, the role of TGF-β1 in MSCs therapy awaiting further in-depth investigation.

Drp1 is a core component of the mitochondrial fission machinery and the increased Drp1-GTPase activity is the key response to induce mitochondrial fragmentation, which is finely tuned by post-translational modifications [60]. Among a variety of post-translational modifications, Drp1 phosphorylation at the Ser-637/616 site plays a pivotal role in the regulation of Drp1-GTPase activity, which modulates Drp1 translocation to the mitochondria [36, 42]. In this work, we found that rSTC1 treatment inhibited the phosphorylation of Drp1-ser616 in a time course-dependent manner, whereas Drp1-ser637 was not prominently altered. In view of the aforementioned performance of MSCs-secreted STC1 on mitochondrial network reprogramming, we raised a question about whether there exists kinase(s) mediated the STC1-Drp1 pathway. Based on the sequence analysis surrounding Ser616, ERK1/2 was presumed to directly regulate Drp1, because the consensus sequence motif of ERK1/2 substrates is identical with that of Drp1 surrounding Ser616. In support of this view, a recent study showed that mitochondrial fission induced by phosphorylation of Drp1 at S616 is a critical event in HG-induced cardiomyocyte hypertrophy by ERK1/2 activation [33]. Using pharmacological and genetic manipulation, we clearly demonstrated that ERK1/2 is a crucial kinase mediating mitochondrial fission by promoting phosphorylation of Ser616 on Drp1 under HG conditions, and STC1-mediated ERK1/2 inactivation reprograms mitochondrial morphology in parallel with improved mitochondrial fitness in endothelial cells. Notably, the importance of this phosphorylation is underscored by the fact that ectopic expression of Drp1-S616D abrogated the beneficial effect of ERK1/2 inactivation on mitochondrial morphology and function improvement. Thus, the STC1-ERK1/2-Drp1 axis is essential for MSCs to orchestrate mitochondrial dynamics remodeling in endothelial cells during HG exposure. However, we cannot eliminate the possibility of involvement of other kinase(s) such as glycogen synthase kinase 3β in high glucose–induced Drp1 Ser616 phosphorylation and mitochondrial fragmentation [42], and do not exclude that other STC1-dependent pathways, independent of Drp1 phosphorylation, may also play important roles in the STC1-mediated maintenance of mitochondrial homeostasis under diabetic conditions.

Numerous studies have described the therapeutic effects of MSCs in vascular medicine, including ischemic diseases, spinal cord injury, and diabetic vasculopathy. For example, MSCs are reported to be able to restore vascularization and blood flow in ischemic stroke [61] and diabetic hindlimb ischemia [62]. Co-transplantation of human MSCs with porcine islets was reported to have a greater cellular insulin content and increased glucose-stimulated insulin secretion in a diabetic mouse model due to earlier islet vascularization and improved islet engraftment [17]. Furthermore, our previous study also indicated that intravenous infusion of MSCs can ameliorate diabetic endothelial dysfunction by promoting mitophagy [19]. The mechanisms by which MSCs exert their therapeutic effects are multifaceted. These therapeutic effects were initially explained by cell replacement or by empowering the regenerative capacity of in situ cells; however, the engraftment rate of MSCs is very low since exogenous MSCs are usually trapped in the lungs and rapidly destroyed by the host immune system [63]. Indeed, the paradoxical observations have been explained by a later finding, in which intravenous injection of MSCs improved myocardial infarction through producing TSG6, which was induced when MSCs were trapped in the lung [64]. These observations suggest that the benefit of MSCs transplantation occurred through the cells’ paracrine activity, rather than through their limited differentiation potentials. Studies over the past few years have demonstrated that MSCs can orchestrate tissue regeneration and homeostasis through the release of various mediators, including immunosuppressive molecules, growth factors, exosomes, chemokines, complement components, and various metabolites [65]. For example, MSC-derived extracellular vesicles can mitigate endothelial cell senescence and stimulate angiogenesis through miR-146a/Src [66]. Furthermore, in the acute lung injury model, MSCs were found to ameliorate endothelial permeability and lung inflammation through paracrine hepatocyte growth factor or Ang-1 mRNA in MSC microvesicles [67, 68]. Beyond that, MSCs have proven to regulate the lesion microenvironment through cell-cell and cell-microenvironment communications. A recent study found that MSCs can promote vascularization and nerve tissue repair in spinal cord injury treatment, based on crosstalk between the vascular endothelial cells and MSCs in the lesion microenvironment [69]. In particular, in vitro and in vivo studies report that MSCs rescue ischemic injury through the delivery of their own mitochondria to endothelial cells through tunneling nanotubes [70]. A similar result has also been reported in the model of acute lung injury [71]. More recently, emerging evidences indicated that MSCs still generate therapeutic effects even when they are dying, especially undergoing apoptosis. In the myocardial infarction model, MSCs released apoptotic bodies were reported to enhance angiogenesis and improve cardiac functional recovery *via* regulating autophagy in the recipient endothelial cells [72]. A study in cutaneous wound healing demonstrated that apoptotic MSCs showed higher inflammatory regulatory abilities, whereas the inhibition of apoptosis led to decreased inflammatory regulatory ability and attenuated their therapeutic effects [73]. Similarly, MSC apoptosis and subsequent efferocytosis are required in animal models of lung injury and inflammation[74]. These observations may answer the longstanding question of how MSCs mediate therapeutic effects that persist beyond their survival. It is plausible that this mechanism is also employed by MSCs to execute their reparative actions in scenarios of endothelial injury, functioning either in conjunction with or parallel to paracrine factors.

In conclusion, our findings demonstrate that MSCs exert vascular protective properties by inhibiting excessive mitochondrial fission mediated by Drp1 in diabetic states. In addition, STC1 may be the principal paracrine factor released by MSCs, which regulates mitochondrial dynamics remodeling through inhibiting ERK1/2-Drp1 axis and, consequently, preventing mtROS overproduction, inflammatory, apoptosis and resultant endothelial dysfunction.

## Supplementary information

Supplemental Information

The original Western blot bands

checklist

## Data Availability

The data underlying this article are available in its online supplementary material.
