# Supplementary material for: MSCs ameliorates hyperglycemia-induced endothelial injury through modulation of mitochondrial dynamics
Source: Cell Death Dis. 2025 Nov 17;16(1):832. doi: 10.1038/s41419-025-08175-x (PMC12624087; doi:10.1038/s41419-025-08175-x)
Supplement: Supplementary file 1 — Supplemental Information [file 41419_2025_8175_MOESM1_ESM.docx]

Supporting Information

**MSCs ameliorates hyperglycemia-induced endothelial injury through modulation of mitochondrial dynamics**

Jingjing Wei^1^**^*^**, Ruiwen Mao^2,3^**^*^**, Yao Chen^1^, Kunjie Si^1^, Yao Li^1^, Jiaqi Li^1^, Wuzheng Zhu^1🖂^

**^1^**Hunan International Joint Laboratory of Animal Intestinal Ecology and Health, Laboratory of Animal Nutrition and Human Health, College of Life Sciences, Hunan Normal University, Changsha, 410081, P.R. China

**^2^**National Medical Metabolomics International Collaborative Research Center, Xiangya Hospital, Central South University, Changsha, 410011, P.R. China

**^3^**National Clinical Research Center for Geriatric Disorders, Xiangya Hospital, Central South University, Changsha, 410011, P.R. China

*****Co-first authors that contributed equally to this work.

🖂 Corresponding author: Wuzheng Zhu, E-mail: [zhuwuzheng8@163.com](mailto:zhuwuzheng8@163.com)

Hunan International Joint Laboratory of Animal Intestinal Ecology and Health, Laboratory of Animal Nutrition and Human Health, College of Life Sciences, Hunan Normal University, Changsha, 410081, P.R. China

**Figure S1**


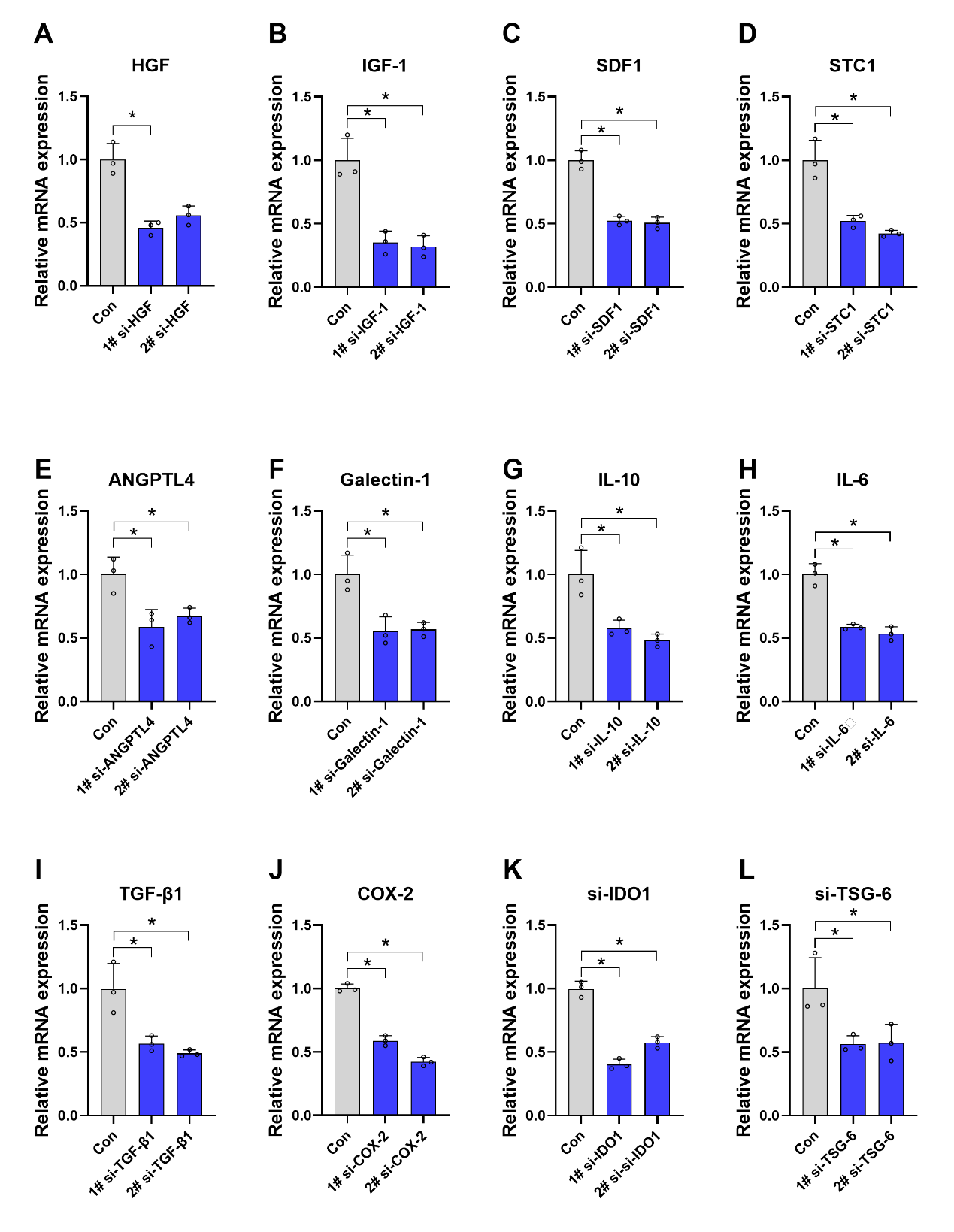


**Figure S1. The gene-silencing effect of siRNAs used in this study. (A-L)** RT-PCR analysis showing the knockdown efficiency of secretory-protein-encoding genes in MSCs at the mRNA level. mRNA expression was expressed as the fold change relative to the value of control cells. Results were shown from three independent experiments (*n* = 3 independent experiments). Data are shown as means ± SD. **P <* 0.05. Data were analysed using one-way ANOVA with Tukey’s *post hoc* test.

**Figure S2**


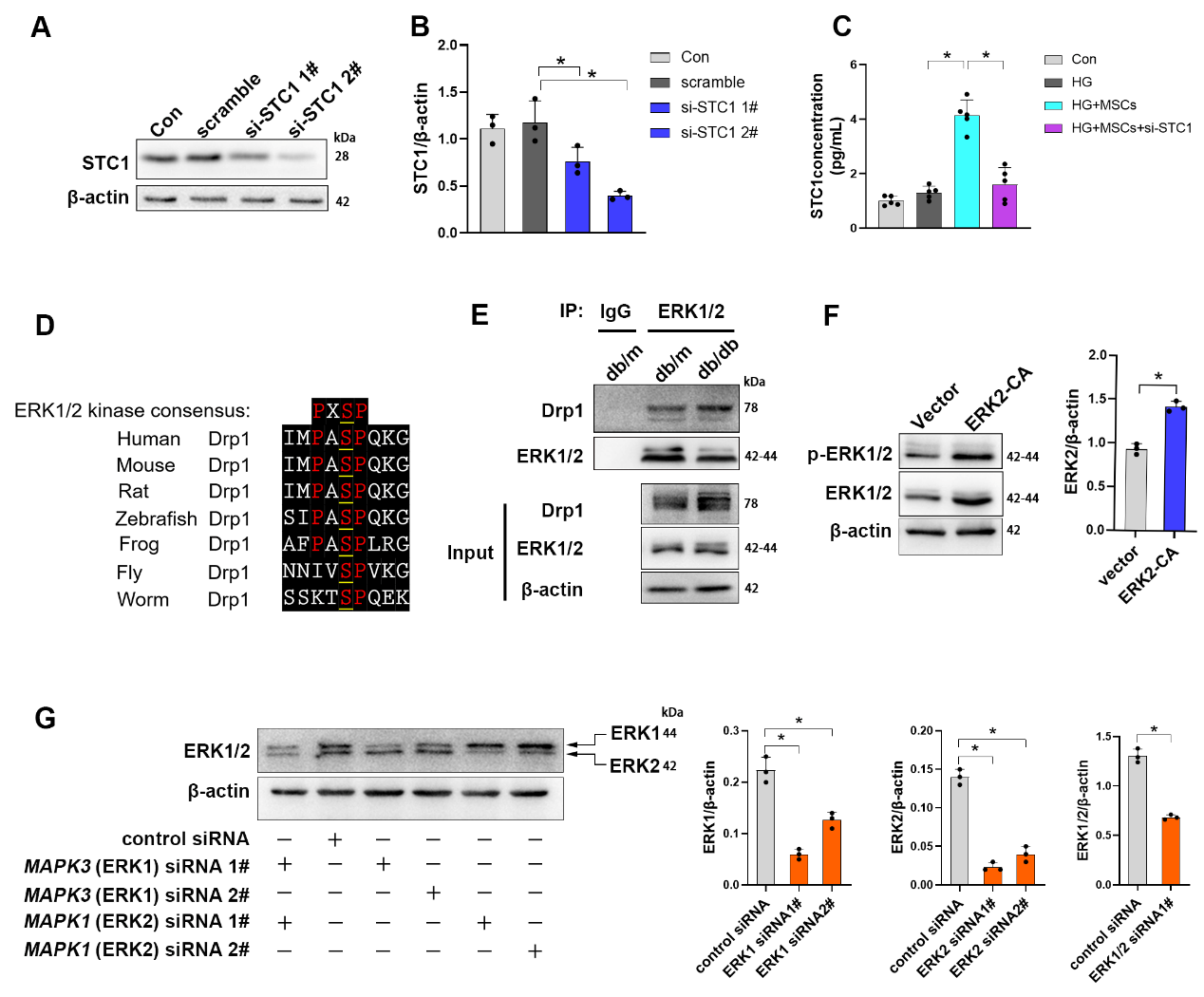


**Figure S2. (A, B)** Representative Western blots and bar graph of STC1 in MSCs transfected with either scramble or si-STC1 (*n* = 3 independent experiments). **(C)** STC1 secretion in supernatant of HUVECs alone or co-cultured with MSCs (*n* = 5 independent experiments). **(D)** Alignment of the consensus Erk1/2 target sequence with amino acids of human Drp1 (isoform 1) and the corresponding sequence from the indicated species. **(E)** Immunoprecipitation (IP) experiment showing the interaction between ERK1/2 and Drp1 in diabetic aortas tissues. **(F)** Representative Western blots (left panel) and bar graph of ERK2 (right panel) in HUVECs cells transfected with either empty vector or constitutive active ERK2 (ERK2-CA) (*n* = 3 independent experiments). **(G)** Expression of total ERK1/2 after siRNA transfection by Western blot. Relative density of ERK1/2 was compared to that of β-actin (*n* = 3 independent experiments). Data are shown as means ± SD. **P <* 0.05. Student’s *t*-test (unpaired) for the comparisons of two groups, and one-way ANOVA with Tukey’s *post hoc* test if more than two groups were compared.

**Figure S3**


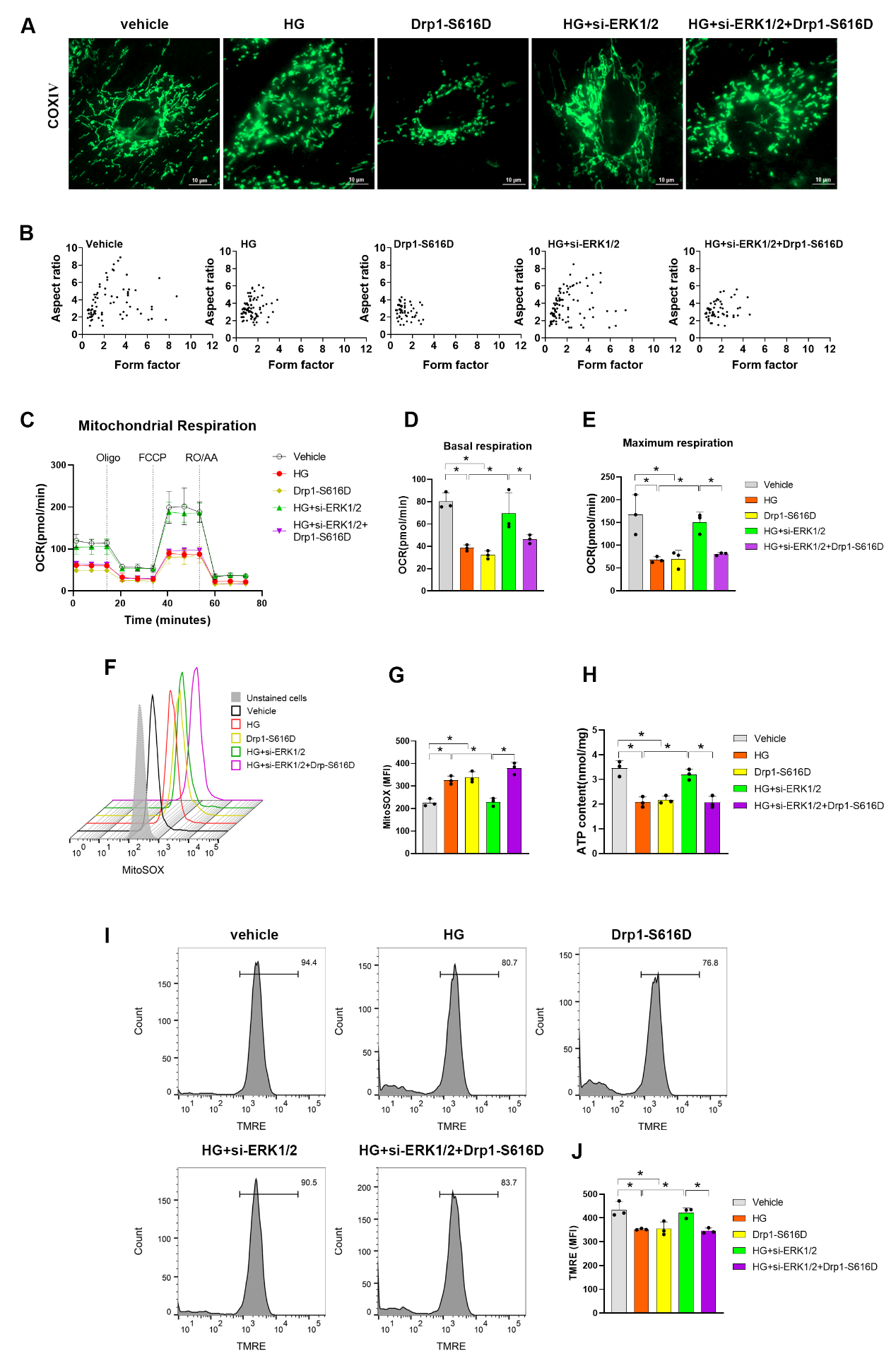


**Figure S3. ERK1/2 mediated Drp1-ser616 phosphorylation promotes alteration of mitochondrial morphology and subsequent mitochondrial compromise under diabetic condition. (A)** Representative images of immunofluorescence analysis of COXIV-labelled mitochondria in HUVECs. **(B)** Aspect ratio and form factor were analyzed for each group (*n* = 60-100 cells/group). (**C-E)** Mitochondrial respiration analysis in HUVECs using the Seahorse platform, and the basal and maximal mitochondrial oxygen consumption rates (OCRs) were quantified (*n* = 3 technical replicates). **(F, G)** HUVECs were loaded with MitoSOX, and analyzed by flow cytometry (*n* = 3 independent experiments). **H** ATP levels were quantiﬁed in HUVECs (*n* = 3 independent experiments). **(I, J)** Mitochondrial membrane potential (ΔΨm) was assayed by using the TMRM probe and determined by flow cytometry (*n* = 3 independent experiments). Data are shown as means ± SD. **P <* 0.05. Data were analysed using one-way ANOVA with Tukey’s *post hoc* test.

**Figure S4**


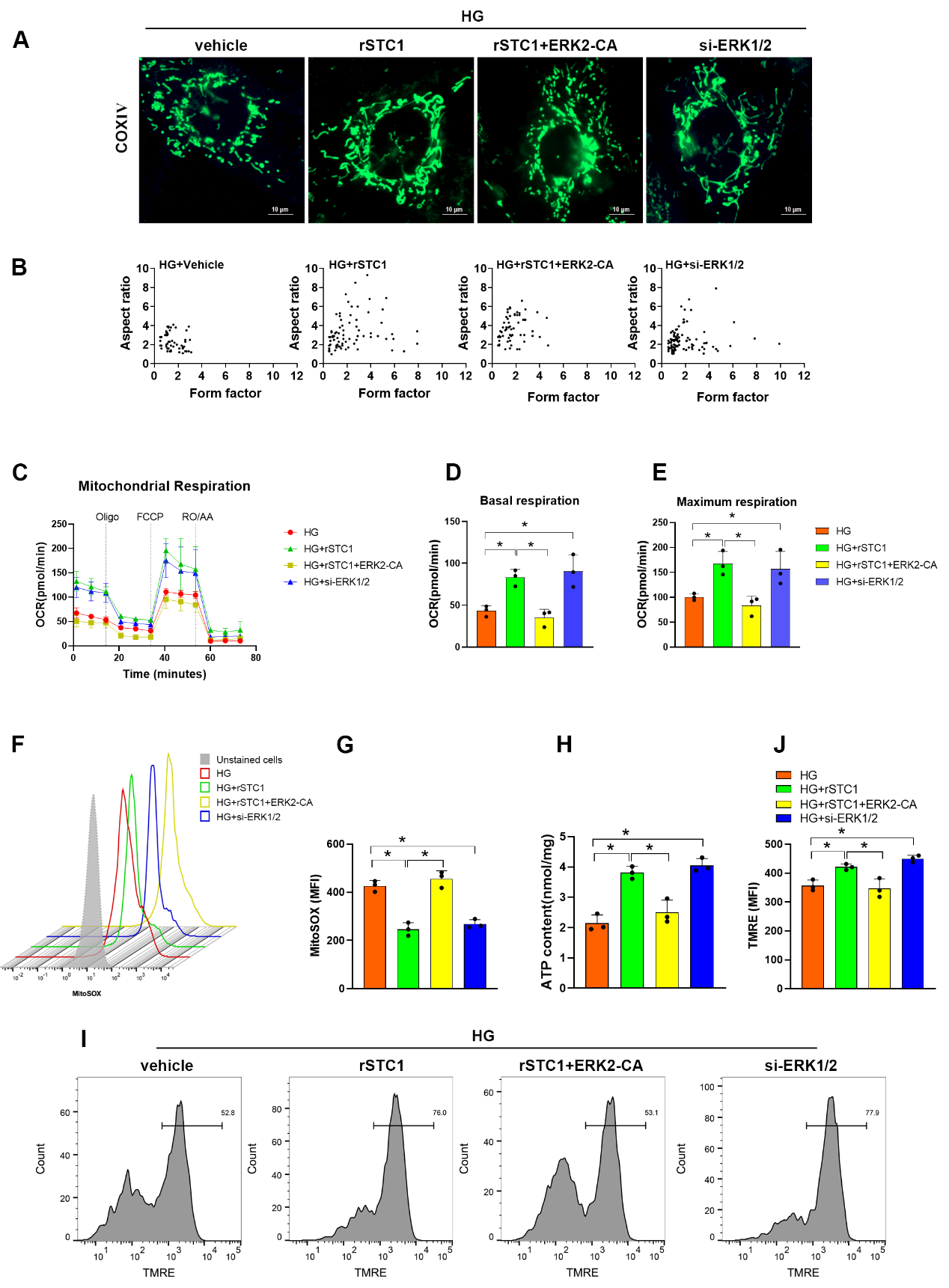


**Figure S4. rSTC1 suppress HG induced mitochondrial fragmentation and mitochondrial dysfunction in a ERK1/2 dependent way. (A)** Representative images of mitochondrial morphology in HUVECs. Mitochondria were labeled using an anti-COXIV antibody. **(B)** Aspect ratio and form factor were quantified for each group (*n* = 60-100 cells/group). **(C-E)** Mitochondrial respiration analysis in HUVECs using the Seahorse platform, and the basal and maximal mitochondrial oxygen consumption rates (OCRs) are quantified (*n* = 3 technical replicates). **(F, G)** HUVECs were loaded with MitoSOX, and analyzed by flow cytometry (*n* = 3 independent experiments). **(H)** ATP levels were quantiﬁed in HUVECs (*n* = 3 independent experiments). **(I, J)** Mitochondrial membrane potential (ΔΨm) was assayed by using the TMRM probe and determined by flow cytometry (*n* = 3 independent experiments). Data are shown as means ± SD. **P <* 0.05. Data were analysed using one-way ANOVA with Tukey’s *post hoc* test.

**Figure S5**


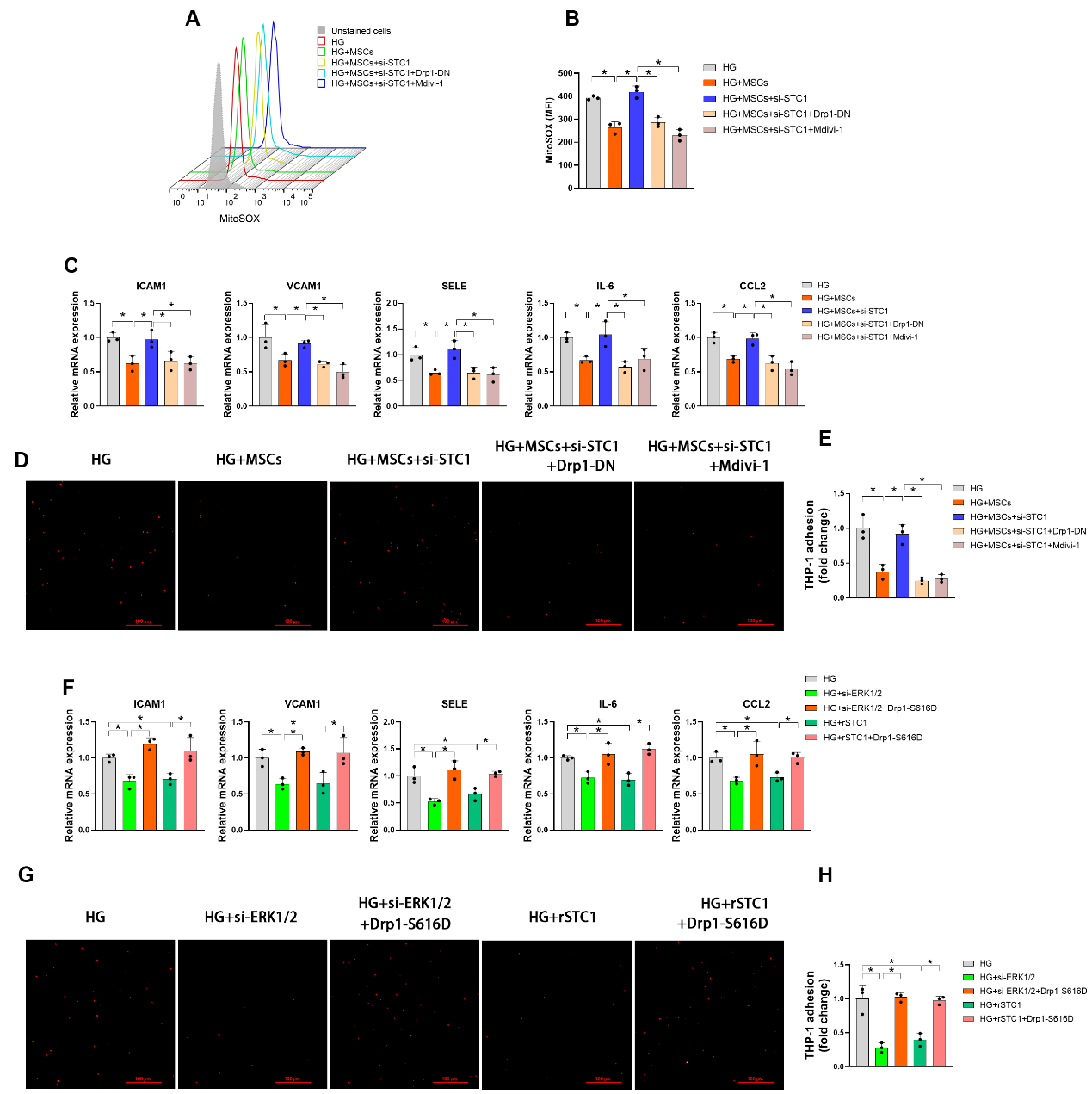


**Figure S5. STC1-ERK1/2-Drp1 axis is essential for MSCs to prevent HG-induced proinflammatory phenotype**. **(A, B)** HUVECs were loaded with MitoSOX, and mitochondrial ROS was analyzed by flow cytometry (*n* = 3 independent experiments). **(C)** mRNA expression of inflammatory adhesion molecules was measured in HUVECs (*n* = 3 independent experiments). **(D, E)** Representative images and corresponding quantitation showed the adhesion of monocytes to the lawn of HUVECs (*n* = 3 independent experiments). **(F)** mRNA expression of inflammatory adhesion molecules of different groups in HUVECs under HG conditions (*n* = 3 independent experiments). **(G, H)** Representative images and corresponding quantitation showed the adhesion of monocytes to the lawn of HUVECs in different groups (*n* = 3 independent experiments). Data are shown as means ± SD. **P <* 0.05. Data were analysed using one-way ANOVA with Tukey’s *post hoc* test.

**Figure S6**


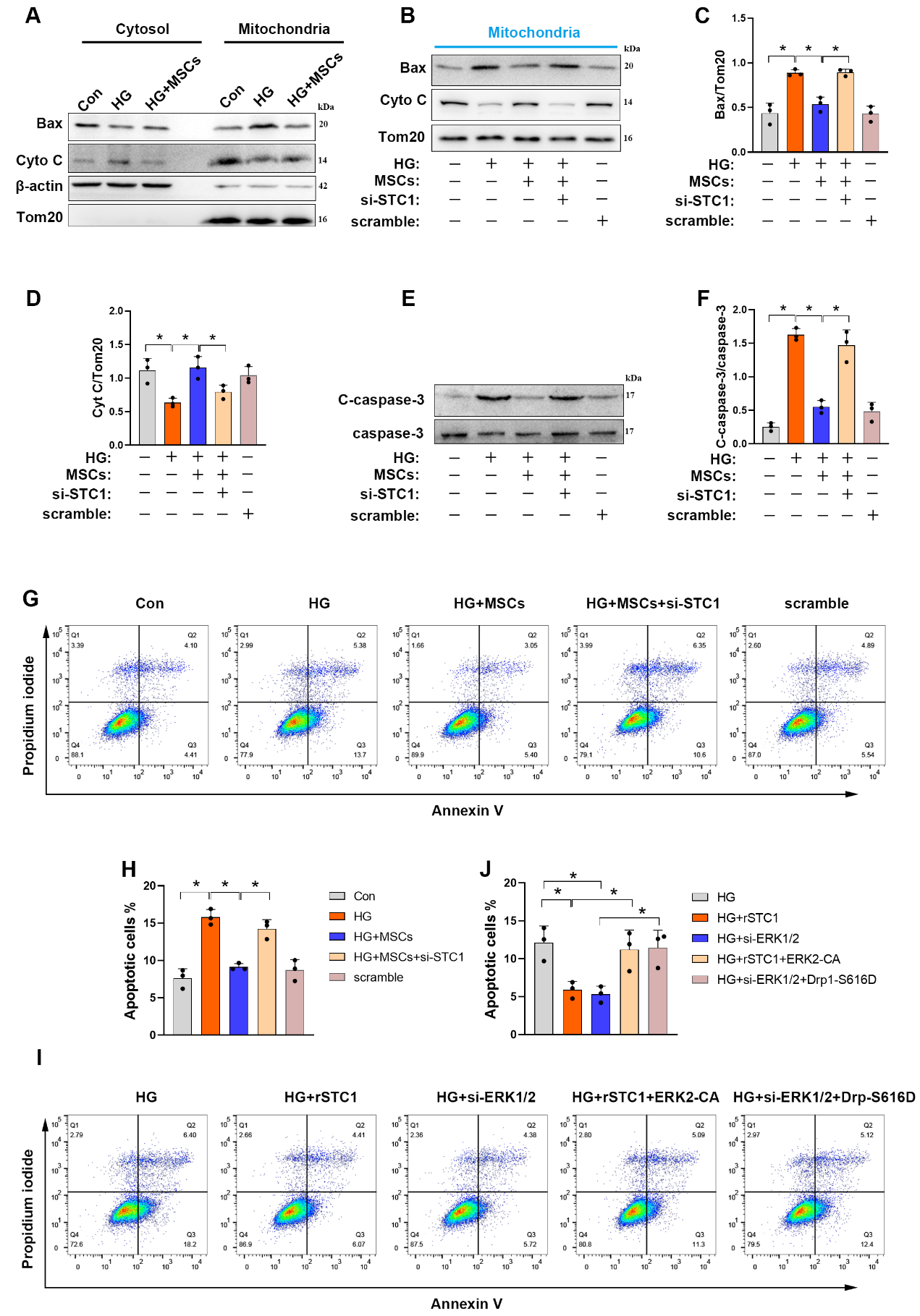


**Figure S6. MSCs suppress HG-induced endothelial apoptosis by STC1-dependent mitochondrial dynamics remodeling. (A)** Cytoplasmic and mitochondrial fractions from HUVECs lysates were analyzed by Western blots, the fractions were probed for cytochrome C and Bax. **(B-D)** Representative Western blots and densitometry analysis of mitochondrial Bax and cytochrome C (Cyto C) in HUVECs (*n* = 3 independent experiments). **(E, F)** Western blots of whole-cell lysates probed for C-caspase-3 and caspase-3 (*n* = 3 independent experiments). **(G-J)** The apoptotic ratios of HUVECs in different groups were determined by flow cytometry (*n* = 3 independent experiments). Data are shown as means ± SD. **P <* 0.05. Data were analysed using one-way ANOVA with Tukey’s *post hoc* test.

**Figure S7**


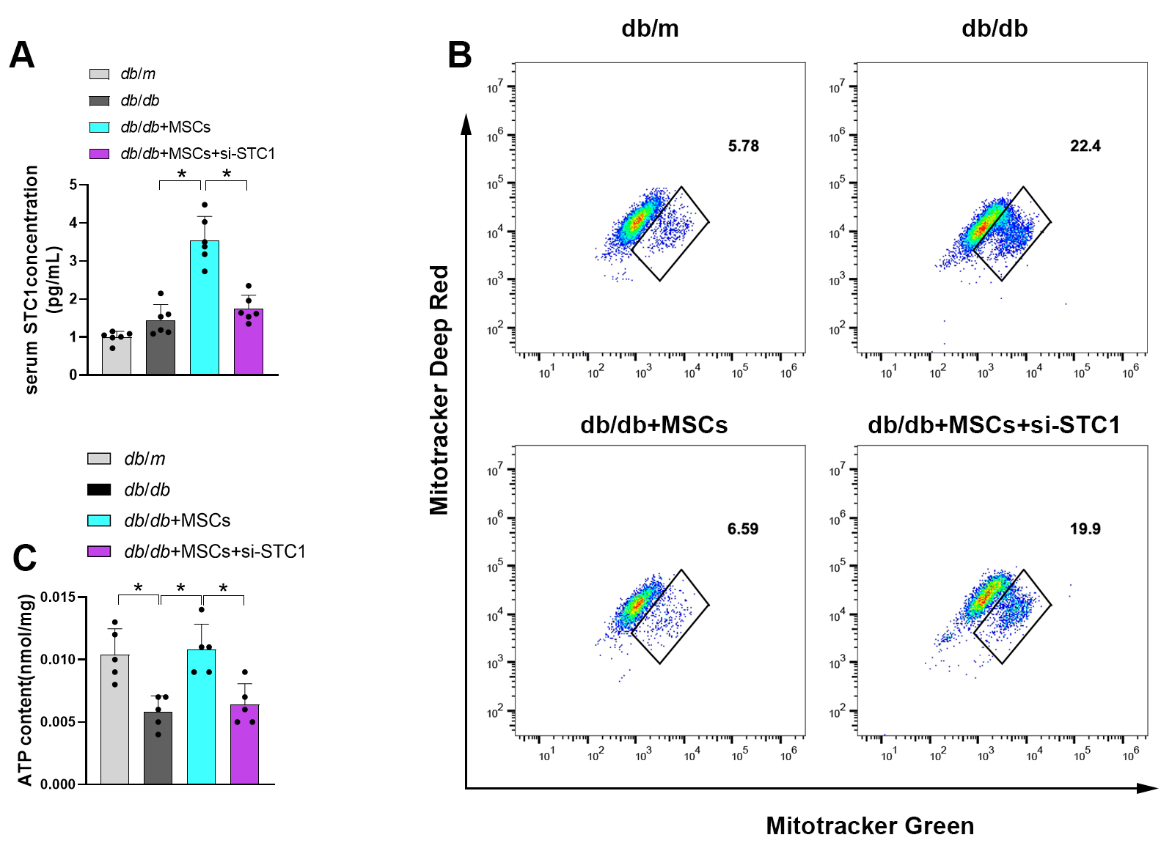


**Figure S7. MSCs treatment improves mitochondrial fitness in the diabetic mice. (A)** The level of STC1 detected by ELISA in mouse serum from different groups (*n* = 6 mice/group). **(B)** Mitochondrial status is analyzed by flow cytometry in isolated aortic mitochondria labelled with MitoTracker Green and MitoTracker Deep Red, gates represent cells with damaged mitochondria. **(C)** ATP levels were quantified in aortic tissues (*n* = 5 independent experiments). Data are shown as means ± SD. **P <* 0.05. Data were analysed using one-way ANOVA with Tukey’s *post hoc* test.

**Figure S8**


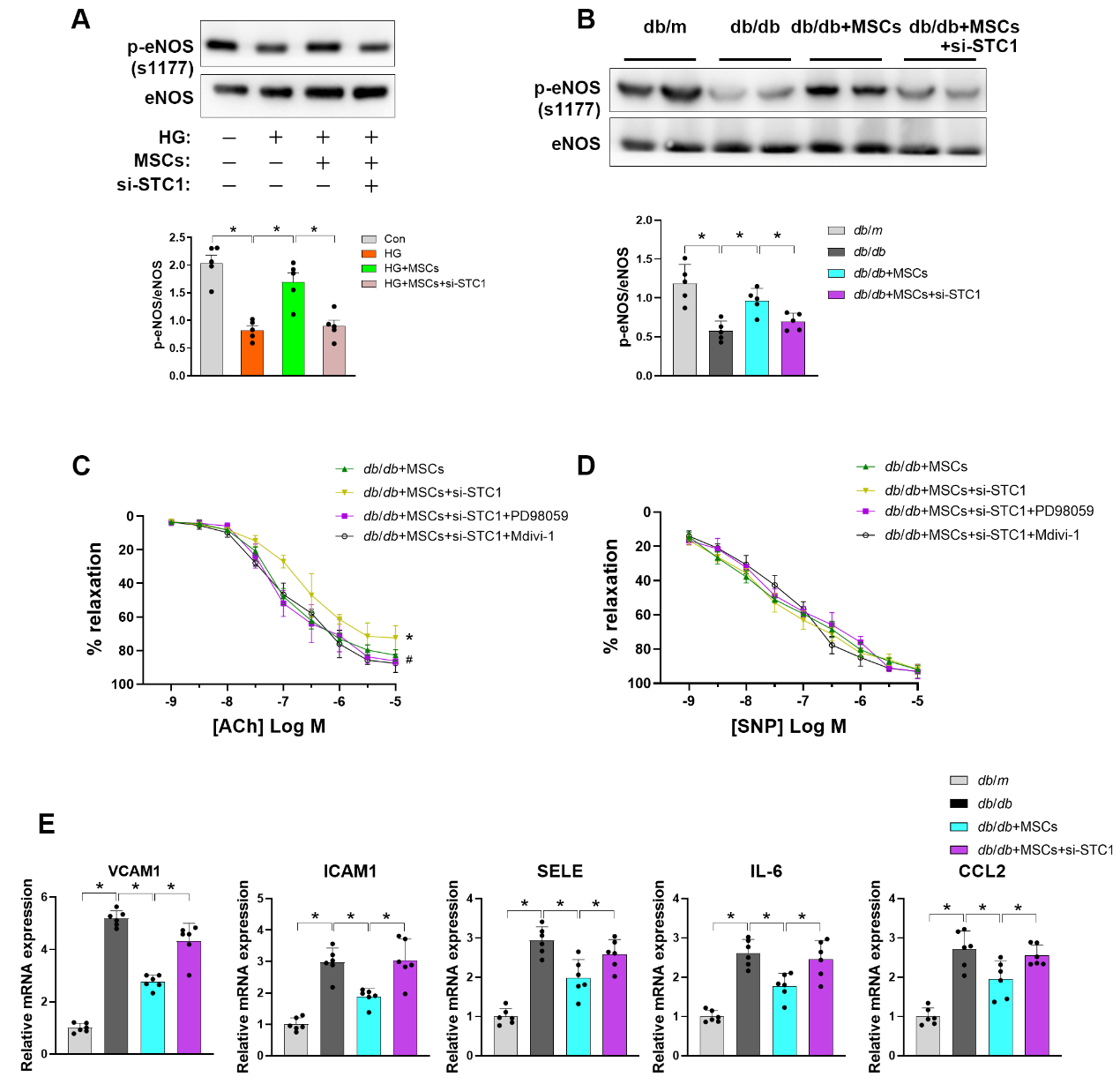


**Figure S8. MSCs secrete STC1 to attenuate endothelial dysfunction in diabetic mice by inhibition of ERK1/2-Drp1 signaling. (A)** Representative Western blots and densitometry analysis of p-eNOS in HUVECs upon HG insults when cocultured with MSCs transfected with STC1 siRNA (n = 5 independent experiments). Data are shown as means ± SD. **P <* 0.05. **(B)** Representative Western blots and densitometric analysis of p-eNOS expression in aorta of *db*/*m* and *db*/*db* mice given saline or MSCs transfected with scramble siRNA or with STC1 siRNA (*n* = 6 mice/group). Data are shown as means ± SD. **P <* 0.05. **(C,** **D)** Acetylcholine (Ach)-induced endothelium-dependent and sodium nitroprusside (SNP)-induced endothelium-independent vasorelaxation in mice aorta precontracted with norepinephrine. Data are shown as means ± SD. **P* < 0.05 vs. *db*/*db*+MSCs, *^#^P* < 0.05 vs. *db*/*db*+MSCs+si-STC1 (*n* = 6 mice/group). **(E)** mRNA expression of vascular proinflammatory genes *VCAM1*, *ICAM1*, *SELE*, *CCL2*, and *IL-6* in mice aortic tissues (*n* = 6 mice/group). Data are shown as means ± SD. **P <* 0.05. Data were analysed using one-way ANOVA with Tukey’s *post hoc* test.

**Supplementary Table 1**

Target sequences of siRNAs used in the siRNA screen

| **Target gene** | **RNA oligo sequences Sense (5′→3′) Antisense (5′→3′)** |
| --- | --- |
| 1# ERK1 | CUGAAUUGUAUCAUCAACAUG |
|  | UGUUGAUGAUACAAUUCAGGU |
| 2# ERK1 | CGUGCUCCACCGAGAUCUAAA |
|  | UAGAUCUCGGUGGAGCACGUU |
| 1# ERK2 | CAAGAAGACCUGAAUUGUAUA |
|  | UACAAUUCAGGUCUUCUUGUG |
| 2# ERK2 | CGAGCAAAUGAAAGAUGUAUA |
|  | UACAUCUUUCAUUUGCUCGAU |
| 1# HGF | CAAUGUGCUAAUAGAUGUACU |
|  | UACAUCUAUUAGCACAUUGGU |
| 2# HGF | AGUGAAUACUGCAGACCAAUG |
|  | UUGGUCUGCAGUAUUCACUUU |
| 1# IGF-1 | CGAAGUCUCAGAGAAGGAAAG |
|  | UUCCUUCUCUGAGACUUCGUG |
| 2# IGF-1 | CAGCAGUCUUCCAACCCAAUU |
|  | UUGGGUUGGAAGACUGCUGAU |
| 1# SDF1 | GAAGAACAACAACAGACAAGU |
|  | UUGUCUGUUGUUGUUCUUCAG |
| 2# SDF1 | GCUGAAGAACAACAACAGACA |
|  | UCUGUUGUUGUUCUUCAGCCG |
| 1# STC1 | GGAUGUAUGACAUCUGUAAAU |
|  | UUACAGAUGUCAUACAUCCCA |
| 2# STC1 | CAGUCAGCACAAUCAGAGACA |
|  | UCUCUGAUUGUGCUGACUGUG |
| 1# ANGPTL4 | GCGAAUUCAGCAUCUGCAAAG |
|  | UUGCAGAUGCUGAAUUCGCAG |
| 2# ANGPTL4 | GAACAGCAGGAUCCAGCAACU |
|  | UUGCUGGAUCCUGCUGUUCUG |
| 1# Galectin-1 | GACGGUGACUUCAAGAUCAAA |
|  | UGAUCUUGAAGUCACCGUCAG |
| 2# Galectin-1 | CCAUCGUGUGCAACAGCAAGG |
|  | UUGCUGUUGCACACGAUGGUG |
| 1# IL-10 | GCUGGACAACUUGUUGUUAAA |
|  | UAACAACAAGUUGUCCAGCUG |
| 2# IL-10 | UGACAAUGAAGAUACGAAACU |
|  | UUUCGUAUCUUCAUUGUCAUG |
| 1# IL-6 | GAACGAAUUGACAAACAAAUU |
|  | UUUGUUUGUCAAUUCGUUCUG |
| 2# IL-6 | GGCAAAGAAUCUAGAUGCAAU |
|  | UGCAUCUAGAUUCUUUGCCUU |
| 1# TGF-β1 | GCAUAUAUAUGUUCUUCAACA |
|  | UUGAAGAACAUAUAUAUGCUG |
| 2# TGF-β1 | CACUGCAAGUGGACAUCAACG |
|  | UUGAUGUCCACUUGCAGUGUG |
| 1# IDO1 | GGACAAUCAGUAAAGAGUACC |
|  | UACUCUUUACUGAUUGUCCAG |
| 2# IDO1 | GGAGAAUAAGACCUCUGAAGA |
|  | UUCAGAGGUCUUAUUCUCCUU |
| 1# COX-2 | GAGCAGUUGUUCCAGACAAGC |
|  | UUGUCUGGAACAACUGCUCAU |
| 2# COX-2 | CAGUAUAAGUGCGAUUGUACC |
|  | UACAAUCGCACUUAUACUGGU |
| 1# TSG-6 | GAUCAUCUUAAUUUACUUAUU |
|  | UAAGUAAAUUAAGAUGAUCAU |
| 2# TSG-6 | GGCAUUAUUGAUUAUGGAAUC |
|  | UUCCAUAAUCAAUAAUGCCAG |
| scramble | UUCUCCGAACGUGUCACGUTT |
|  | ACGUGACACGUUCGGAGAATT |

**Supplementary Table 2**

Real time qPCR primers for detection of human genes

| **Gene Symbol** | **Primer sequences 5ˊto 3ˊ** | **Species** |
| --- | --- | --- |
| VCAM1 | F: CAGTAAGGCAGGCTGTAAAAGA R: TGGAGCTGGTAGACCCTCG | Human |
| ICAM1 | F: TTGGGCATAGAGACCCCGTT  R: GCACATTGCTCAGTTCATACACC | Human |
| IL6 | F: CCTGAACCTTCCAAAGATGGC R: TTCACCAGGCAAGTCTCCTCA | Human |
| CCL2 | F: CAGCCAGATGCAATCAATGCC R: TGGAATCCTGAACCCACTTCT | Human |
| SELE | F: TGTGGGTCTGGGTAGGAACC R: AGCTGTGTAGCATAGGGCAAG | Human |
| GAPDH | F: CCACTCCTCCACCTTTGAC R: ACCCTGTTGCTGTAGCCA | Human |
| HGF | F: CGACAGTGTTTCCCTTCTCG R: ATTGAGAACCTGTTTGCGTTTCT | Human |
| IGF-1 | F: CAAGCCTGCCAAGTCAGCTC R: TAGTTCTTGTTTCCTGCACTCCCT | Human |
| ANGPTL4 | F: CAAGGCTCAGAACAGCAGGA R: CCCCTGAGGCTGGATTTCAA | Human |
| IL-10 | F: GGAGGTGATGCCCCAAGCTGA R: AATCGATGACAGCGCCGTAGC | Human |
| IL-6 | F: TCTCCACAAGCGCCTTCG R: CTCAGGGCTGAGATGCCG | Human |
| TGF-β1 | F: CAAGCAGAGTACACACAGCAT R: TGCTCCACTTTTAACTTGAGCC | Human |
| IDO1 | F: GCCTGTGTGAAAGCTCTGGTC R: CCTCCAGTTCCTTTGGCTTCC | Human |
| TSG-6 | F:TGTCTGTGCTGCTGGATGGAT R:TGTGGGTTGTAGCAATAGGCAT | Human |
| SDF1 | F:AGCCAACGTCAAGCATCTCAA R:AATCCACTTTAGCTTCGGGTCAA | Human |
| STC1 | F:AGGTGCAGGAAGAGTGCTACA R:GACGACCTCAGTGATGGCTT | Human |
| COX-2 | F:TGCCTGATGATTGCCCGACT R:TGAAAGCTGGCCCTCGCTTA | Human |

**Supplementary Table 3**

Real time qPCR primers for detection of mouse genes

| **Gene Symbol** | **Primer sequences 5ˊto 3ˊ** | **Species** |
| --- | --- | --- |
| VCAM1 | F: GTTCCAGCGAGGGTCTACC  R: AACTCTTGGCAAACATTAGGTGT | Mouse |
| ICAM1 | F: GTGATGCTCAGGTATCCATCCA R: CACAGTTCTCAAAGCACAGCG | Mouse |
| SELE | F: ATGCCTCGCGCTTTCTCTC R: GTAGTCCCGCTGACAGTATGC | Mouse |
| CCL2 | F: TTAAAAACCTGGATCGGAACCAA R: GCATTAGCTTCAGATTTACGGGT | Mouse |
| IL6 | F: TTCAGCCCTTGCTTGCCTC R: ACACTTTTACTCCGAAGTCGGT | Mouse |
| GAPDH | F: AGGTCGGTGTGAACGGATTTG R: TGTAGACCATGTAGTTGAGGTCA | Mouse |
