# Supplementary figures and images for: MSCs ameliorates hyperglycemia-induced endothelial injury through modulation of mitochondrial dynamics
Source: Cell Death Dis. 2025 Nov 17;16(1):832. doi: 10.1038/s41419-025-08175-x (PMC12624087; doi:10.1038/s41419-025-08175-x)

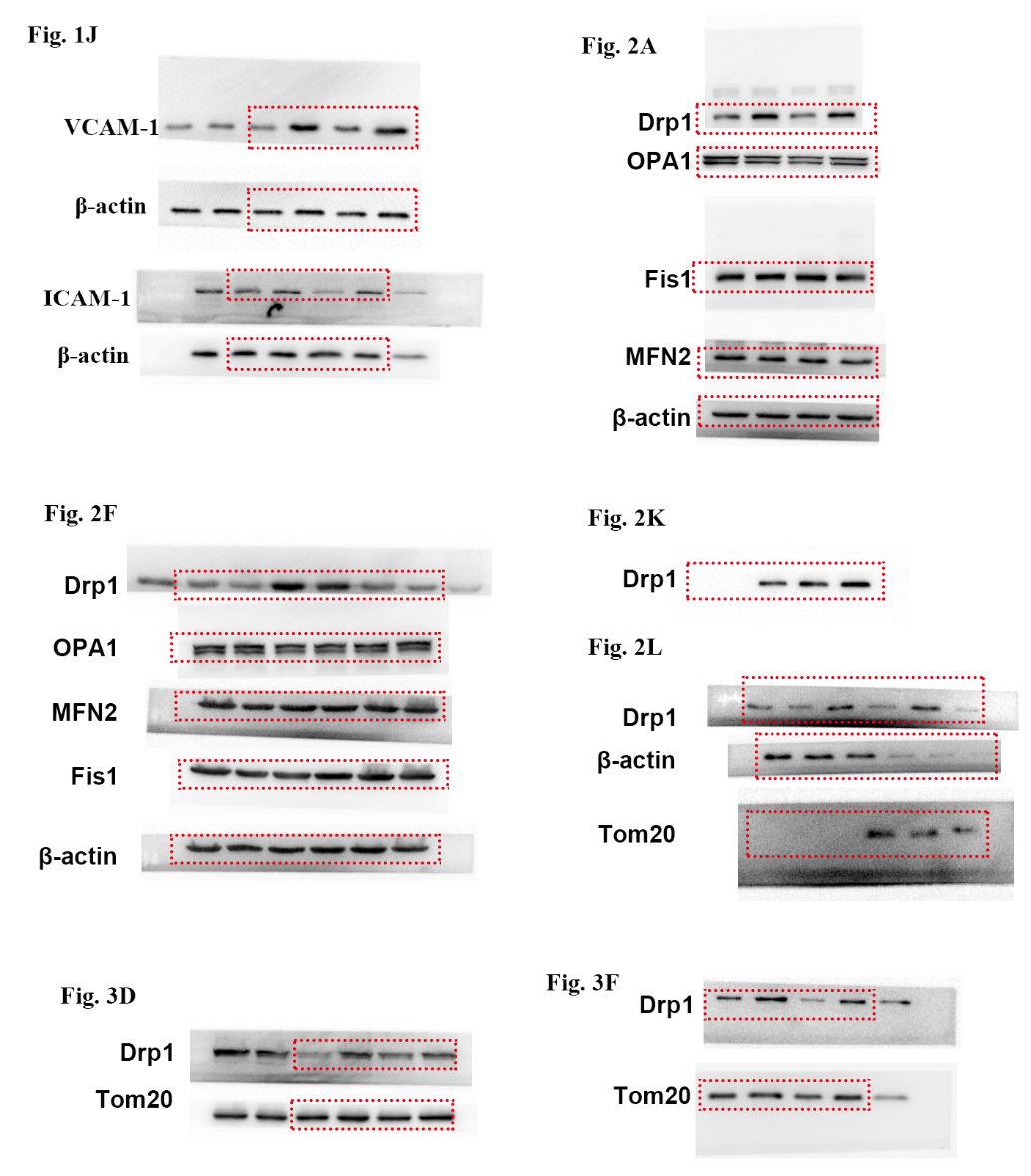


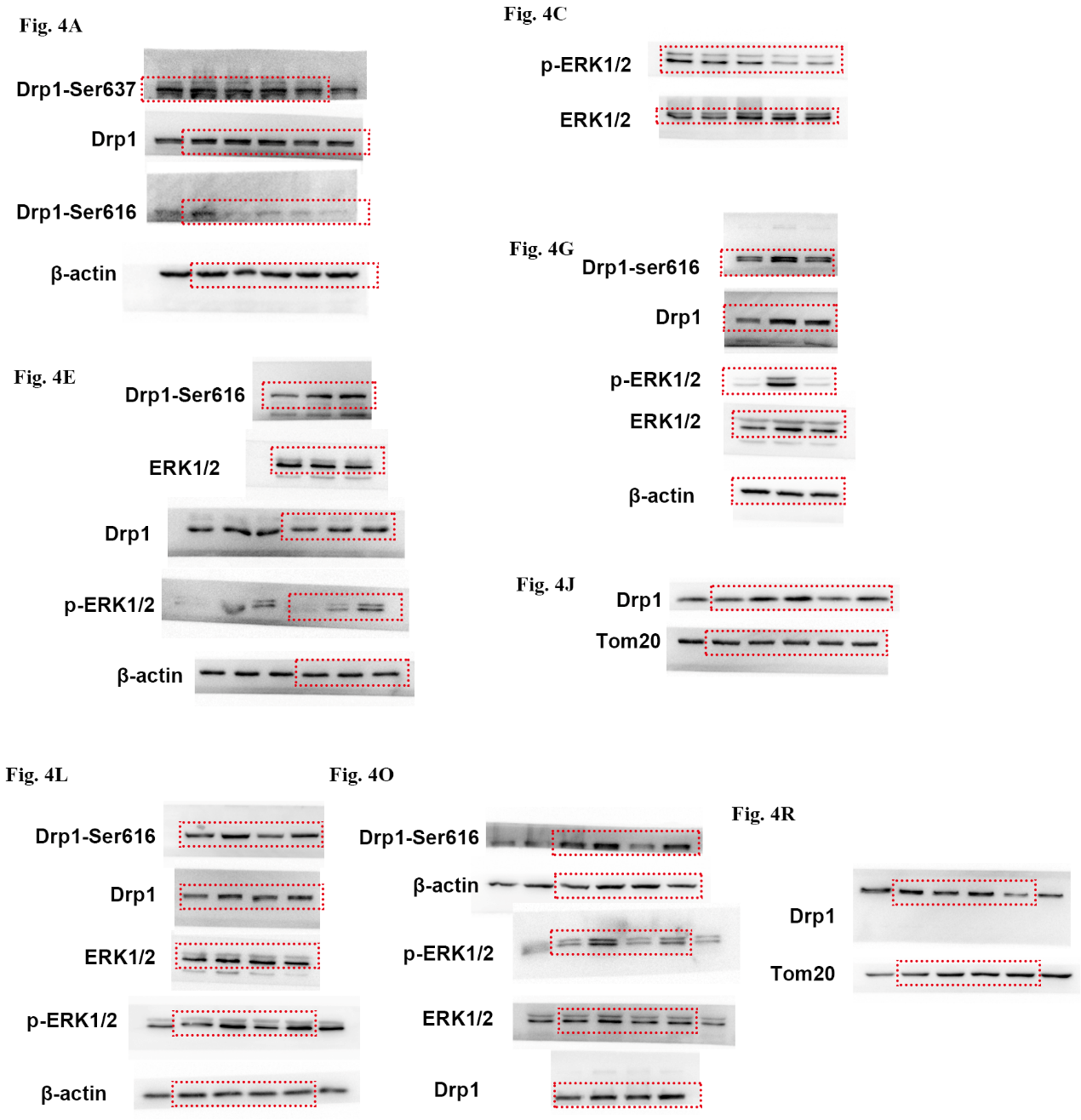


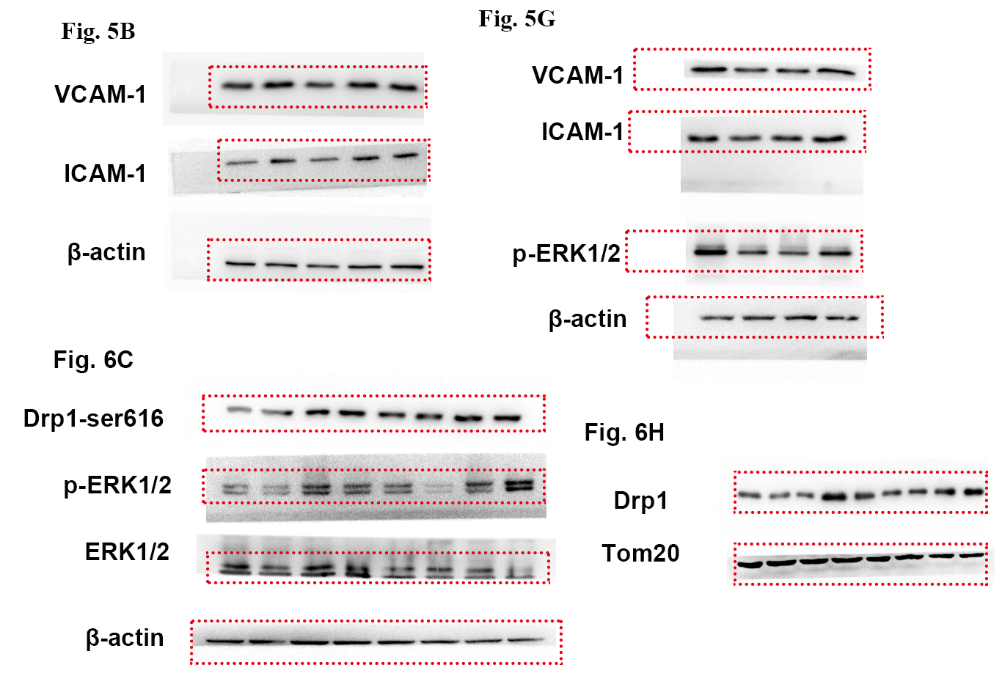


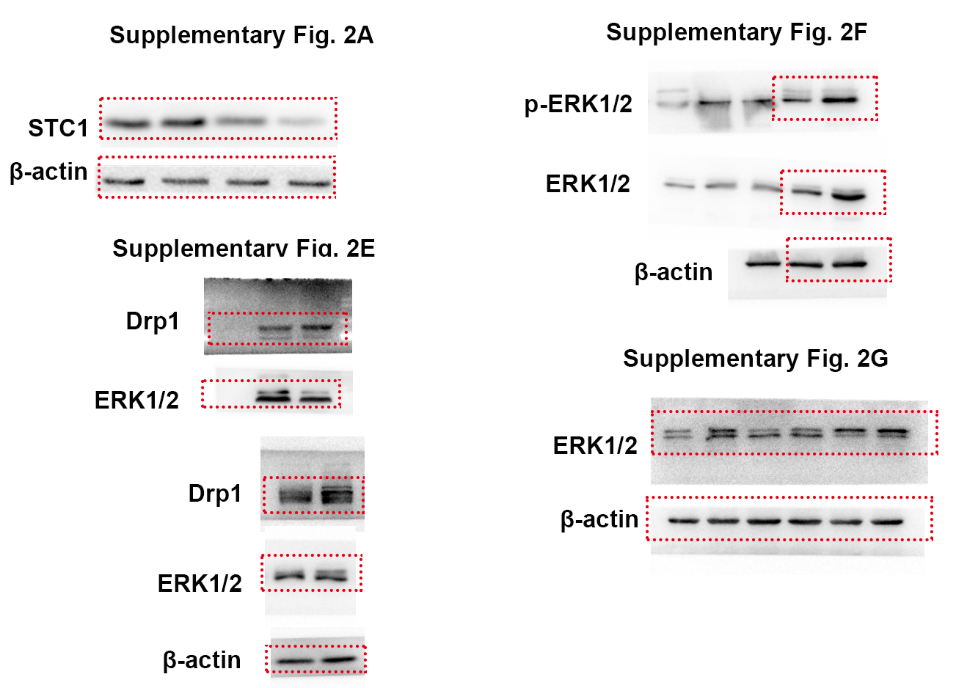


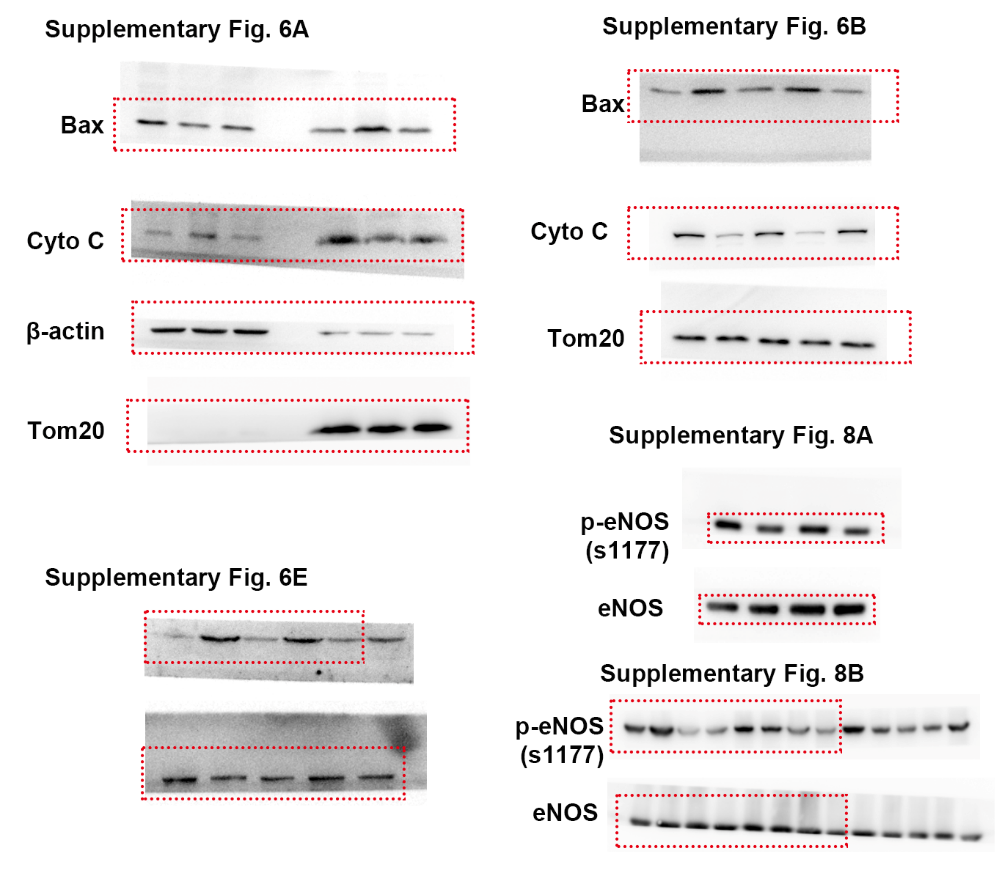

Supplement: Supplementary file 2 — The original Western blot bands [file 41419_2025_8175_MOESM2_ESM.docx]
